# Nitric Oxide Generated by Tumor-Associated Macrophages Is Responsible for Cancer Resistance to Cisplatin and Correlated With Syntaxin 4 and Acid Sphingomyelinase Inhibition

**DOI:** 10.3389/fimmu.2018.01186

**Published:** 2018-05-29

**Authors:** Cristiana Perrotta, Davide Cervia, Ilaria Di Renzo, Claudia Moscheni, Maria Teresa Bassi, Lara Campana, Cristina Martelli, Elisabetta Catalani, Matteo Giovarelli, Silvia Zecchini, Marco Coazzoli, Annalisa Capobianco, Luisa Ottobrini, Giovanni Lucignani, Patrizia Rosa, Patrizia Rovere-Querini, Clara De Palma, Emilio Clementi

**Affiliations:** ^1^Department of Biomedical and Clinical Sciences “L. Sacco”, Università degli Studi di Milano, Milan, Italy; ^2^Department for Innovation in Biological, Agro-Food and Forest Systems, Università degli Studi della Tuscia, Viterbo, Italy; ^3^“Eugenio Medea” Scientific Institute, Bosisio Parini, Italy; ^4^Division of Immunology, Transplantation and Infectious Diseases, San Raffaele Scientific Institute, Milan, Italy; ^5^Medical Research Council Centre for Inflammation Research, University of Edinburgh, Edinburgh, United Kingdom; ^6^Department of Pathophysiology and Transplantation, Università degli Studi di Milano, Milan, Italy; ^7^Unit of Clinical Pharmacology, University Hospital “L. Sacco”-ASST Fatebenefratelli Sacco, Department of Biomedical and Clinical Sciences, CNR-Institute of Neuroscience, Università degli Studi di Milano, Milan, Italy; ^8^CNR-Institute for Molecular Bioimaging and Physiology, Milan, Italy; ^9^Department of Health Sciences, Università degli Studi di Milano, Milan, Italy; ^10^Department of Medical Biotechnologies and Translational Medicine Pharmacology, CNR-Institute of Neuroscience, Università degli Studi di Milano, Milan, Italy; ^11^Università Vita-Salute San Raffaele, Milan, Italy

**Keywords:** tumor-associated macrophages, nitric oxide, acid sphingomyelinase, syntaxin 4, cisplatin resistance

## Abstract

Tumor microenvironment is fundamental for cancer progression and chemoresistance. Among stromal cells tumor-associated macrophages (TAMs) represent the largest population of infiltrating inflammatory cells in malignant tumors, promoting their growth, invasion, and immune evasion. M2-polarized TAMs are endowed with the nitric oxide (NO)-generating enzyme inducible nitric oxide synthase (iNOS). NO has divergent effects on tumors, since it can either stimulate tumor cells growth or promote their death depending on the source of it; likewise the role of iNOS in cancer differs depending on the cell type. The role of NO generated by TAMs has not been investigated. Using different tumor models *in vitro* and *in vivo* we found that NO generated by iNOS of M2-polarized TAMs is able to protect tumor cells from apoptosis induced by the chemotherapeutic agent cisplatin (CDDP). Here, we demonstrate that the protective effect of NO depends on the inhibition of acid sphingomyelinase (A-SMase), which is activated by CDDP in a pathway involving the death receptor CD95. Mechanistic insights indicate that NO actions occur *via* generation of cyclic GMP and activation of protein kinase G (PKG), inducing phosphorylation of syntaxin 4 (synt4), a SNARE protein responsible for A-SMase trafficking and activation. Noteworthy, phosphorylation of synt4 at serine 78 by PKG is responsible for the proteasome-dependent degradation of synt4, which limits the CDDP-induced exposure of A-SMase to the plasma membrane of tumor cells. This inhibits the cytotoxic mechanism of CDDP reducing A-SMase-triggered apoptosis. This is the first demonstration that endogenous NO system is a key mechanism through which TAMs protect tumor cells from chemotherapeutic drug-induced apoptosis. The identification of the pathway responsible for A-SMase activity downregulation in tumors leading to chemoresistance warrants further investigations as a means to identify new anti-cancer molecules capable of specifically inhibiting synt4 degradation.

## Introduction

Chemotherapeutic agent cisplatin (CDDP) is a widely used and very effective chemotherapeutic drug that induces apoptosis of cancer cells in solid tumors (1). However, CDDP has a major drawback; while patients in the first line of treatment usually respond to it, tumors develop resistance over time (2, 3), mostly because of their ability to escape the apoptogenic effects of the drug (4). Recent studies have revealed a more complex scenario in which tumor resistance is also acquired through changes in the tumor milieu both in the cells composing the tumor microenvironment, as for instance immune cells, and in the soluble factors they release locally (5, 6).

Inside the tumor microenvironment, tumor-associated macrophages (TAMs) have been demonstrated to be fundamental for cancer progression (7). In the tumor mass, these cells are subjected to a variety of stimuli that change their features. Initially, TAMs are in the M1 (classically activated) state, that is pro-inflammatory and anti-tumorigenic; as tumors progress they evolve to the M2 (alternatively activated) state, a pro-tumoral phenotype that triggers tissue remodeling as well as immune-suppression (8–10). TAMs are predominantly polarized as M2 macrophages in tumor of high grade associated with poor prognosis, and promote proliferation, survival, and motility of cancer cells (8, 10–17).

Tumor-associated macrophages are competent to express inducible nitric oxide (NO) synthase (iNOS) and generate the gaseous messenger NO (18–20). At low, physiological levels NO displays cytoprotective properties while it is cytotoxic when produced at high concentrations (19, 21–24). NO cytoprotection in cancer has been linked to the inhibition of the sphingomyelin metabolizing enzyme acid sphingomyelinase (A-SMase) whose activation is triggered by death receptors, i.e., CD95 and TNFRI, and chemotherapeutic drugs such as CDDP (25–27). We have previously found that an important determinant of A-SMase activity is the target-SNARE protein syntaxin 4 (synt4), known to be involved in several exocytosis events, such as antibody secretion as well as secretory pathway of CTLs and GLUT4 transport on the plasma membrane (28–31). In U373 human glioma cells stimulated by CD95, we showed that synt4 presides over the vesicular trafficking of A-SMase allowing its exposure to the plasma membrane and hence its activation (28, 32). Intriguingly syntaxins expression and activity, including that of synt4, have been linked to tumor progression through mechanisms still unknown (33–37).

We thus decided to investigate the molecular relationship between NO, A-SMase and synt4 and the role they play in the development of tumor resistance to CDDP. By *in vivo* and *in vitro* experiments using human and murine cell models we found that NO, produced at low levels by iNOS from M2-like TAMs, protects cancer cells from CDDP-induced apoptosis leading to chemoresistance. NO phosphorylates synt4 promoting its proteasomal degradation in a pathway involving generation of cyclic GMP (cGMP) and activation of protein kinase G (PKG). Synt4 downregulation in turn inhibits CDDP-induced A-SMase traslocation to the plasma membrane and its activation, thus blocking CDDP-apoptogenic action against tumor cells. We thus define for the first time a mechanism whereby NO regulation of synt4 causes chemoresistance to CDDP leading to the control of sphingolipid metabolism. The identification of this system in the tumor microenvironment contributes to shed light on the role of TAMs in the pathophysiology of cancer.

## Materials and Methods

### Immunofluorescence

Immunohistochemical staining was performed on tissue microarray (TMA) samples obtained from US Biomax (#GL805L, Rockville, MD, USA), using published protocols (38–41). The paraffin-embedded tissue arrays were baked at 60°C for 30 min, then were dewaxed in xylene for 10 min twice and finally were rehydrated through a series of alcohol solutions (Sigma-Aldrich, Saint Louis, MO, USA) (100, 95, and 70%, respectively ethanol) to water. For antigen retrieval, the TMA samples were heated to 95°C for 15 min in 0.01 M sodium citrate buffer (Sigma-Aldrich, Saint Louis, MO, USA) at pH 6.0. After returning to room temperature, the TMA samples were rinsed with 0.1 M phosphate buffer (PB) and subsequently blocked with 10% of normal goat serum (NGS; Thermo Fisher Scientific, Waltham, MA, USA) in PB for 30 min at room temperature. For double-immunofluorescence staining, TMA samples were incubated with rabbit anti-iNOS antibody (1:500 dilution; #ab178945, Abcam, Cambridge, UK) and mouse anti-CD206 antibody (1:100 dilution; #MCA2155; Bio-Rad, Hercules, CA, USA) in PB containing 0.1% Triton X-100 overnight at 4°C. Following washes in PB, TMA was incubated with the appropriate Alexa Fluor secondary antibodies (Life Technologies-Thermo Fisher Scientific, Waltham, MA, USA) in PB containing 0.1% Triton X-100 for 1.5 h at room temperature. Finally, the TMA was coverslipped with Fluoroshield Mounting Medium containing DAPI (Abcam, Cambridge, UK). Images were acquired using a 40× objective by a Zeiss LSM 710 confocal microscope (Carl Zeiss, Oberkochen, Germany).

### Human and Mouse M2-Polarized Macrophage Preparation

Human polarized macrophages were propagated as described (42): briefly, human monocytes, derived from peripheral blood of healthy donors using sequential Ficoll-Paque PLUS (GE Healthcare, Chicago, IL, USA) and 46% Percol (GE Healthcare, Chicago, IL, USA) density gradients, were cultured for 5 days in X-VIVO 15 (Cambrex Bio Science, Verviers, Belgium) supplemented with 1% human serum (BioWhittaker, Walkersville, MD, USA) (culture medium) and recombinant human rhM-CSF (R&D Systems, Minneapolis, MN, USA) (100 ng/ml) at a density of 2.5 × 10^5^/cm^2^. Cells were then cultured for an additional 2 days in X-VIVO 15 supplemented with 1% HS and recombinant human rhIL-4 (R&D Systems, Minneapolis, MN, USA) (10 ng/ml) for M2 polarization. Macrophage differentiation was validated by the expression of HLA A, B, and C (PE Mouse Anti-Human HLA-ABC antibody, Clone G46-2.6, BD Bioscience, San Jose, CA, USA) by flow cytometry (Gallios, Beckman-Coulter, Brea, CA, USA) (20). The M2 phenotype was confirmed by the expression of the M2 markers CD163 and CD206 (mouse anti-human CD163 antibody, clone GHI/61, and mouse anti-human CD206, Clone 19.2, BD Bioscience, San Jose, CA, USA) and by the low expression of the M1 marker CD14 (mouse anti-human CD14, clone M5E2, BD Bioscience, San Jose, CA, USA).

For murine M2 macrophages preparation, bone marrow cell suspensions were isolated by flushing femurs and tibias of 8- to 12-week-old C57BL/6 wt and iNOS^−/−^ mice with MEM alpha supplemented with 10% fetal bovine serum (FBS) (Euroclone, Milan, Italy), 2 mM glutamine (Euroclone, Milan, Italy), 100 U/ml penicillin, and 100 U/ml streptomycin (Euroclone, Milan, Italy) (complete MEM alpha). Aggregates were dislodged by gentle pipetting, and debris was removed by passaging the suspension through a 40-µm nylon cell strainer. Cells were washed twice with medium, and seeded on ultra-low attachment surface plates. Cells were cultured in complete MEM alpha supplemented with recombinant mouse rhM-CSF (Miltenyi Biotec, Bergisch Gladbach, Germany) (100 ng/ml) and cultured in a humidified incubator at 37 C and 5% CO_2_ for 7 days. On days 3 and 5, cells were amplified and medium was refreshed. Cells were then cultured for additional 4 days in complete MEM alpha supplemented with rhM-CSF (10 ng/ml) and recombinant mouse rhIL-4 (Miltenyi Biotec, Bergisch Gladbach, Germany) (10 ng/ml) for M2 polarization. Flow cytometry analysis demonstrated macrophage differentiation (F4/80) (anti-mouse F4/80, clone BM8, #123110, Biolegend, San Diego, CA) and the M2 phenotype (CD206) (anti-mouse CD206 antibody, clone C068C2, #141704, Biolegend, San Diego, CA, USA) (43). The expression of M2 markers was confirmed by real-time PCR (Figure S3D in Supplementary Material) (43).

### Cell Cultures and Pharmacological Treatments *In Vitro*

U373 human glioma (American Type Culture Collection), GL261 murine glioma (a kind gift of Dr. Serena Pellegatta, “Carlo Besta” Neurological Institute, Milan, Italy), and Lewis lung carcinoma (LLC) cells (American Type Culture Collection) were routinely grown in Dulbecco’s Modified Eagle Medium (DMEM) (Euroclone, Milan, Italy), supplemented with 10% FBS (Euroclone, Milan, Italy), 2 mM glutamine (Euroclone, Milan, Italy), 100 U/ml penicillin (Euroclone, Milan, Italy), and 100 U/ml streptomycin (Euroclone, Milan, Italy) at 37°C, 5% CO_2_.

All pharmacological treatments on U373 and GL261 cells were carried out in the culture medium. In brief, incubations with Nω-Nitro-l-arginine methyl ester hydrochloride (l-NAME; 2 mM) (Sigma-Aldrich, Saint Louis, MO, USA) or (z)-1-[2-(2-aminoethyl)-N-(2-ammonioethyl)amino]diazen-1-ium-1,2-diolate (DETA-NO; 20 µM) (Calbiochem—Merck, Darmstadt, Germany) or 8Br-cGMP (3 mM) (Sigma-Aldrich, Saint Louis, MO, USA) or human neutralizing anti-CD95 antibody ZB4 (500 ng/ml) were performed for 1 h before CDDP (50 µg/ml for U373 and 20 µg/ml for GL261) (Teva Pharmaceuticals Europe B.V., Amsterdam, The Netherlands) administration (44). H-[1,2,4]oxadiazolo[4,3-α]quinoxalin-1-one (ODQ; 1 µM) (Enzo Life Sciences, Farmingdale, New York, NY, USA) was added to the cell culture 15 min prior to DETA-NO administration. KT5823 (1 µM) (Calbiochem—Merck, Darmstadt, Germany) was added to the cell culture 15 min prior to DETA-NO administration. Incubation with MG132 (10 µM) (Calbiochem—Merck, Darmstadt, Germany) was performed for 2 h before 8Br-cGMP and CDDP administration.

### Viability Assay

Cell viability of human and mouse glioma cells in the presence of CDDP at increasing concentrations for 24 h was evaluated by MTT analysis (40, 45, 46). MTT (Sigma-Aldrich, Saint Louis, MO, USA) absorbance was quantified spectrophotometrically using a Glomax Multi Detection System microplate reader (Promega, Madison, WI, USA).

### Proliferation Assay

U373 and GL261 cell proliferation after 24 h of treatment with CDDP was assessed by measuring the serial halving of cell fluorescence intensity *via* flow cytometry (45). The CytoTrack Cell Proliferation Assays (CytoTrack Green; Bio-Rad, Hercules, CA, USA) was used, according to the manufacturer’s protocol. Fluorescence was analyzed by Gallios Flow Cytometer (Beckman-Coulter, Brea, CA, USA) and the software FCS Express 4 (*De Novo* System, Portland, OR, USA). The proliferation index, defined as the average number of cells that an initial cell became, was calculated using FCS Express software.

### Transwell Co-Cultures

M2-polarized human or mouse macrophages (2 × 10^6^) from wt or iNOS^−/−^ mice were plated on the upper compartment of 0.4-µm pore size transwell plates in 0.1 ml of DMEM complete medium, while respectively U373 human or GL261 murine glioma cells (0.3 × 10^6^) were plated in the lower chamber. Co-cultures were incubated for 24 h after which cells in the lower chamber received pharmacological treatments. Precisely, U373 cells were treated as follows: CDDP (50 µg/ml); l-NAME (2 mM) pre-incubated 1 h before CDDP; DETA-NO (20 µM) and l-NAME pre-incubated 1 h before CDDP administration. GL261 cells co-cultured with wt M2 macrophages were treated as follows: CDDP (20 µg/ml); l-NAME (2 mM) pre-incubated 1 h before CDDP; DETA-NO (20 µM) and l-NAME pre-incubated 1 h before CDDP administration. GL261 cells co-cultured with iNOS^−/−^ M2 macrophages were treated with CDDP (20 µg/ml). After additionally 24 h, U373 or GL261 cells and murine wt and iNOS^−/−^ M2 macrophages were harvested and used for evaluation of apoptosis.

### Apoptosis Detection

Phosphatidylserine exposure on the outer leaflet of the plasma membrane was detected by analysis of cells stained for 15 min with Alexa Fluor 488-labeled annexin V (1 µg/ml) (Molecular Probes—Thermo Fisher Scientific, Waltham, MA, USA) and propidium iodide (PI, Sigma-Aldrich, Saint Louis, MO, USA) or VivaFix649 (Bio-Rad, Hercules, CA, USA), using a Flow Cytometer (Gallios, Beckman-Coulter, Brea, CA, USA) as described previously (40, 45, 46). For the experiment with the human neutralizing anti-CD95 antibody ZB4 (500 ng/ml) cells were also visualized by brightfield microscopy by using the ZOE Fluorescent Cell Imager (Bio-Rad, Hercules, CA, USA).

### Griess Reaction

Nitric oxide generation in cell culture supernatants of human and murine M2 macrophages and by DETA-NO (20 µM) was assessed by the Griess method to measure nitrites, which are stable breakdown products of NO. Briefly, culture supernatants and DETA-NO were incubated with the Griess reagents I (1% sulphanilamide in 2.5% phosphoric acid) and II (0.1% naphthylenediamine in 2.5% phosphoric acid). The absorbance was read within 5 min at 550 nm and actual concentration calculated using a standard curve with serial dilutions of sodium nitrite.

### Lentiviral Infection

For the infection, 1 × 10^5^ GL261 cells were seeded in 6-well plate and 2 h before infection growing medium was replaced with IMDM medium (Euroclone, Milan, Italy) supplemented with 10% of FBS HyClone (GE Healthcare, Chicago, IL, USA), 1% penicillin/streptomycin, and 1% l-glutamine (infection medium). After 2 h, cells were infected with the vector (pCCL.PGK.luciferase.WPRE-PLW-vector, kindly provided by Dr. Stefano Rivella, The Children’s Hospital of Philadelphia, PA, USA) with a MOI of 5, in presence of polybrene (8 µg/ml) (Sigma-Aldrich, Saint Louis, MO, USA). Cells were washed 16 h later in order to remove all viral particles, then cells were cultured normally. After having been acquired at CCD camera to evaluate luciferase production, cells were implanted in C57BL/6 mice.

### Animals

Female C57BL/6 mice and iNOS^−/−^ mice (B6.129P2-*Nos2^tm1Lau^*/J mice) (6–8 weeks old) were purchased from Charles River Laboratories and Jackson Laboratories, respectively. Null mutation of iNOS was confirmed by PCR using an upstream primer that was common for both wild-type (wt) and mutant DNA (5′-ACATGCAGAATGAGTACCGG-3′), a wt downstream primer (5′-TCAACATCTCCTGGTGGAAC-3′), and a downstream primer for the neomycin cassette (5′-AATATGCGAAGTGGACCTCG-3′). Animals were kept in a regulated environment (23 ± 1°C, 50 ± 5% humidity) with a 12 h light/dark cycle (lights on at 08:00 a.m.) and fed *ad libitum*. All studies were conducted in accordance with the Italian law on animal care N° 116/1992 and the European Communities Council Directive EEC/609/86.

### Orthotopic Brain Tumor Model, Pharmacological Treatment, and Bioluminescence Imaging

Luciferase-infected GL261 cell suspension (2 µl, 1 × 10^8^ cells/ml) in phosphate buffer saline (PBS) was delivered into the right striatum (0.2 µl/min) by stereotactic injection through a glass electrode connected to a Hamilton syringe. The following coordinates were used: antero-posterior = 0; medio-lateral = + 2.5 mm; dorso-ventral = − 3.5 mm.

Nine days after orthotopic implantation of tumor cells into the brain, mice were randomly assigned to receive the following treatments at days 9, 11, and 13: (a) intraperitoneal (i.p.) injection of PBS (control group), (b) i.p. injection of CDDP at 4 mg/kg (CDDP group); (c) i.p. injection of l-NAME at 4 mg/kg (l-NAME group); (d) i.p. injection of CDDP at 4 mg/kg combined with l-NAME at 4 mg/kg (l-NAME + CDDP group) (Figure S4A in Supplementary Material). Mice were sacrificed when they reached IACUC euthanasia criteria, as for instance clinical signs of tumor.

Intracranial tumor growth was monitored at days 9, 14, and 18 using the IVIS Lumina (Xenogen, Caliper Life Science—Perkin Elmer, Waltham, MS, USA). Mice were injected i.p. with d-luciferin (Beetle Luciferin Potassium Salt; Promega, Madison, WI, USA) at 50 mg/kg and then they were placed in the light-tight chamber, and a gray-scale image of the animals was first taken with dimmed light (FOV—Field of View—12.5 cm). After biodistribution time (20 min) photon emission was acquired for 10 min. Mice were acquired before and after each treatment. For co-localization of the bioluminescent photon emission on the animal body, gray-scale and pseudocolor images were merged using the Living Image Software^®^ (Caliper Life Sciences). To compare different mice, all images were scaled after all the acquisitions with the same scale and ROI analysis were performed to quantify luciferase signal intensity within tumor.

### Subcutaneous Tumor Model and Treatment

Lewis lung carcinoma (2 × 10^5^) cells resuspended in PBS (Euroclone, Milan, Italy) were injected into the right flank of mice and allowed to grow. Nine days after tumor cells injection, mice were randomly assigned to receive the following treatments at days 9, 11, and 13: (a) i.p. injection of PBS (control group), (b) a daily i.p. injection of CDDP at 4 mg/kg (CDDP group); (c) i.p. injection of l-NAME at 4 mg/kg (l-NAME group); (d) i.p. injection of CDDP at 4 mg/kg combined with l-NAME at 4 mg/kg (l-NAME + CDDP group) (Figure S4A in Supplementary Material). Tumor growth was monitored every 2–3 days by means of external caliper measurements and volume calculation (length × width^2^/2), until mice reached IACUC euthanasia criteria, as for instance clinical signs of tumor or when tumor size exceeded 10% of body weight (ca. 1,500 mm^3^ tumor volume). When indicated, mice were sacrificed when tumor size reached ca. 500 mm^3^ volume, and tumor collected for further analysis (i.e., TUNEL assay and western blotting).

### TUNEL Assay

Lewis lung carcinoma tumors were collected and fixed in ice cold 4% paraformaldehyde before being rinsed in PBS and cryo-protected overnight in 30% sucrose. Tissues were then embedded in O.C.T. Compound (Sakura, AJAlphen aan den Rijn, The Netherlands) and cut in a CM1850 UV cryostat (Leica Biosystems, Wetzlar, Germany). At least five cryosections (6 µm) were assayed for apoptosis by the TUNEL method (DeadEnd Fluorometric TUNEL System), according to the manufacturer’s protocol (46, 47). Samples were counterstained with DAPI, mounted with Vectashield (Vector Laboratories, Burlingame, CA, USA), and examined using a DMI4000 B automated inverted microscope equipped with a DCF310 digital camera (Leica Microsystems, Wetzlar, Germany). Image acquisition was controlled by the Leica LAS AF software.

### Intratumoral Macrophage Reconstitution and Tumor Treatment

Macrophage depletion was accomplished with injection of clodronate liposome (48). Briefly, 3 days after tumor injection, mice received a first i.p. administration of 200 µl of PBS liposomes (control) (PBS-LIPO) or clodronate liposomes (CL-LIPO; 1 mg/ml). Mice were, then, treated every 2 days with an intratumor injection of 50 µl PBS liposomes (control) or clodronate liposomes (0.25 mg/ml) (Liposoma B.V., Amsterdam, The Netherlands) till day 9 after tumor inoculation when macrophage depletion was assessed in two mice (Figure S4C in Supplementary Material). For macrophage reconstitution, 5 × 10^5^ bone marrow-derived macrophages from wt or iNOS^−/−^ mice were injected intratumor at day 11 and 13 after tumor inoculation. CDDP was i.p. administered at 4 mg/kg every 2 days from day 12 to 16 after tumor injection. Tumor growth was monitored every 2–3 days by means of external caliper measurements and volume calculation (length × width^2^/2), until mice reached IACUC euthanasia criteria, as for instance clinical signs of tumor or when tumor size exceeded 10% of body weight (ca. 1,500 mm^3^ tumor volume).

### Real-Time PCR

The analysis of mRNA expression was performed as previously described (40, 43, 49, 50). Briefly, total RNA from U373 and GL261 cells was extracted with the PureZol RNA Isolation Reagent (Bio-Rad, Hercules, CA, USA), according to the manufacturer’s protocol. First-strand cDNA was generated from 1 µg of total RNA using iScript Reverse Transcription Supermix (Bio-Rad, Hercules, CA, USA). A set of primer pairs (Eurofins Genomics, Milan, Italy) was designed to hybridize to unique regions of the appropriate gene sequence (Table 1). PCR was performed using SsoAdvanced Universal SYBR Green Supermix and the CFX96 Touch Real-Time PCR Detection System (Bio-Rad, Hercules, CA, USA). The fold change was determined relative to the control after normalizing to Rpl32 (internal standard) through the use of the formula 2^−ΔΔCT^.

**Table 1:** Primer pairs designed for real-time PCR analysis.

Gene name	Forward primer sequence	Reverse primer sequence
*Human smpd1*	5′-TGGCTCTATGAAGCGATGGC-3′	5′-TTGAGAGAGATGAGGCGGAGAC-3′
*Murine smpd1*	5′-TGGGACTCCTTTGGATGGG-3′	5′-CGGCGCTATGGCACTGAAT-3′
*Human stx 4*	5′-CGGACAATTCGGCAGACTATT-3′	5′-TTCTGGGGCTCTATGGCCTT-3′
*Murine stx 4*	5′-CCCGGACGACGAGTTCTT-3′	5′-TTTGATCTCCTCTCGCAGGTT-3′
*Murine arg1*	5′-CTCCAAGCCAAAGTCCTTAGA-3′	5′-AGGAGCTGTCATTAGGGACAT-3′
*Murine ccl-2*	5′-AGGTGTCCCAAAGAAGCTGTA-3′	5′-ATGTCTGGACCCATTCCTTCT-3′
*Murine ccl-9*	5′-CCCTCTCCTTCCTCATTCTTACA-3′	5′-AGTCTTGAAAGCCCATGTGAAA-3′
*Murine cd36*	5′-ATGGGCTGTGATCGGAACTG-3′	5′-GTCTTCCCAATAAGCATGTCTCC-3′
*Murine il-10*	5′-GCTCTTACTGACTGGCATGAG-3′	5′-CGCAGCTCTAGGAGCATGTG-3′
*Human rpl32*	5′-TTTAAGCGTAACTGGCGGAAAC-3′	5′-AAACATTGTGAGCGATCTCGG-3′
*Murine rpl32*	5′-TTAAGCGAAACTGGCGGAAAC-3′	5′-TTGTTGCTCCCATAACCGATG-3′

### A-SMase Activity

Acid sphingomyelinase activity was determined by measuring conversion of sphingomyelin to phosphorylcholine in cell homogenates using the Amplex Red Sphingomyelinase Assay Kit (Molecular Probes-Thermo Fisher Scientific, Waltham, MA, USA) according to the two-step standard protocol. In brief, 2 × 10^6^ cells were homogenated with 0.2% Triton X-100 in H_2_O for 15 min at 4°C, sonicated, and incubated overnight at 80°C. For enzymatic activity assay 100 µg of homogenate from each sample were diluited in 100 µl of sodium acetate (50 mM), pH 5.0, and plated in a 96-well microplate. Similarly, a negative control without the enzyme was set up. The first step reaction was started adding 10 µl of the sphingomyelin solution (5 mM) to samples or negative control and incubated at 37°C for 1 h. At this point, two positive controls were prepared diluiting sphingomyelinase from *Bacillus cereus* at the final concentration of 4 U/ml and H_2_O_2_ 10 µM in 100 µl of 1× reaction buffer and adding 10 µl of the sphingomyelin solution (5 mM). The second step reaction was performed adding to samples, and negative and positive controls, 100 µl of the Amplex Red reagent containing 2 U/ml HRP, 0.2 U/ml choline oxidase, and 8 U/ml alkaline phosphatase and incubated for 30 min at 37°C, protected from light. The fluorescence was measured in a fluorescence microplate reader using excitation in the range of 530–560 nm and emission detection at ~590 nm. For each point, background fluorescence was corrected by subtracting the values derived from the no-sphingomyelinase control (40, 51).

### Western Blotting

Cells and resected allografts were homogenized in lysis buffer (50 mM Tris–HCl pH 7.4, 150 mM NaCl, 1 mM EDTA, 1 mM EGTA, 1% Triton X-100, 10% glycerol) supplemented with a cocktail of protease and phosphatase inhibitors (cOmplete and PhosSTOP; Roche Diagnostics, Milan, Italy) and centrifuged at 1,500 × *g* for 5 min at 4°C to discard cellular debris. After separation by SDS-polyacrylamide gel electrophoresis (MiniProtean TGX precast gels and Criterion TGX Stain-free precast gels; Bio-Rad, Hercules, CA, USA), polypeptides were electrophoretically transferred to nitrocellulose filters using a Bio-Rad Trans-Blot Turbo System. The membranes were probed using the following primary antibodies: rabbit anti-A-SMase (custom synthetized, Areta, Gerenzano, Italy) (28, 40, 52, 53), mouse anti-synt4 (for detection human protein), mouse anti-neuronal NOS (nNOS), mouse anti-endothelial NOS (eNOS) (#610439, #610309, and #610297, BD Bioscience, San Jose, CA, USA), rabbit anti-iNOS (#ab178945, Abcam, Cambridge, UK), rabbit anti-synt4 (for detection of murine protein) (#110042 Synaptic System, Goettingen, Germany), mouse anti-GAPDH and anti-vinculin (#G9295, #v4505, Sigma-Aldrich, Saint Louis, MO, USA). After the incubation with the appropriate horseradish peroxidase conjugated secondary antibody (Bio-Rad, Hercules, CA, USA), bands were visualized using the Clarity Western ECL substrate (Bio-Rad, Hercules, CA, USA) exposure to autoradiography Cl-Xposure films (Thermo Fisher Scientific, Waltham, MA, USA) or with a ChemiDoc MP imaging system (Bio-Rad, Hercules, CA, USA). When appropriated, bands were quantified vs the respective loading control (GAPDH, vinculin, or total proteins) for densitometry using the Bio-Rad Image Lab software.

### Cell Surface Biotinylation Assay

U373 cells were stimulated with CDDP in the absence or in the presence of DETA-NO or 8Br-cGMP at the indicated times in culture medium. Stimulation was stopped with ice cold PBS, cells were washed twice with PBS, and then incubated twice with EZ-Link Sulfo-NHS-LC-Biotin (0.5 mg/ml) (Thermo Fisher Scientific, Waltham, MA, USA) in DMEM without serum for 10 min at 4°C. After washing with serum-free DMEM for 10 min and three times with PBS for 5 min at 4°C, cells were solubilized in lysis buffer (10 mM Tris–HCl pH 7.4, 150 mM NaCl, 1 mM EDTA, 0.1% SDS, 1% Triton X-100 with protease inhibitor cocktail) for 30 min at 4°C. Lysates were then centrifuged for 5 min at 1,500 × *g* and streptavidin agarose beads (Thermo Fisher Scientific, Waltham, MA, USA) were added to the supernatant to isolate cell membrane proteins. After incubation of the mixture for 16 h at 4°C, biotin–streptavidin beads complexes were sedimented at 18,000 × *g* for 3 min. The supernatant was conserved as control, and, after two washes with PBS, bead-bound proteins were denatured in Laemmli’s buffer and analyzed by SDS-PAGE followed by western blotting with the anti-A-SMase antibody as described (28, 40). Cell surface exposure of A-SMase was normalized to 25 µg of total cytosolic lysate for each sample.

### Immunoprecipitation

U373 cells were grown to subconfluency on a 10-cm dish and then treated first with MG132 for 2 h and then with 8Br-cGMP for 30 min before lysis. After washing with PBS, cells were scraped with RIPA buffer (50 mM Tris–HCl Ph 7.4, 150 mM NaCl, 1 mM EDTA, 0.1% SDS, 1% NP-40, 1% sodium deoxycholate) containing protease and phosphatase inhibitor cocktails and incubated for 30 min at 4°C. Cell suspension was centrifuged and protein concentration was measured for adjusting protein amount. Lysates were incubated with Agarose-TUBEs (Tandem Ubiquitin Binding Entities Tebu-Bio, Magenta, Italy) for 4 h at 4°C with rotation. After centrifugation, the immunoprecipitate was washed four times with TBS-T. The pellet was resuspended in SDS 1× sample buffer, treated by heating for 10 min at 80°C and subjected to SDS-PAGE followed by western blotting. Anti-synt4 antibody (#610439, BD Bioscience, San Jose, CA, USA) was used at a dilution of 1:1,000.

### Site-Directed Mutagenesis and Transfection

Mutations of the serine residues of synt4 were performed by PCR cloning using the QuikChange site-directed mutagenesis kit (Stratagene—Agilent, Santa Clara, CA, USA) according to the manufacturer’s instructions. As template for the PCR reaction we used the pCMV6-XL4-Synt4 plasmid containing a native form of human synt4 (Origene, Rockville, MD, USA). The primers used for the S to A mutation were as follows:

S78A, 5′-CCCTTCCCGAGGAGGCCATGAAGCAGGAGC-3′;
S208A, 5′-CTCGGCCCGGCACGCTGAGATCCAGCAG-3′;
S216A, 5′-CCAGCAGCTTGAACGCGCTATTCGTGAGCTGCAC-3′;
S247a, 5′-TTGAGAAGAACATCCTGGCCTCAGCGGACTACGTGG-3′.

All transformants were sequenced to verify the integrity of mutations. Transfection of U373 cells (~80% confluence) with the native and the mutated forms of synt4 was carried out using Fugene Tranfection Reagent (Promega, Madison, WI, USA), according the manufacturer’s protocol. Analysis of synt4 expression levels was performed by western blotting 48 h after transfection.

### Statistical Analysis

Statistical significance of raw data between the groups in each experiment was evaluated using unpaired Student’s *t*-test (single comparisons) or one-way ANOVA followed by the Newman–Keuls post-test (multiple comparisons). When data are not normally distributed, the Mann–Whitney test was used. Tumor growth was analyzed using two-way ANOVA, followed by Bonferroni post-test. The GraphPad Prism software package (Graph Software, San Diego, CA, USA) was used. When indicated, data belonging from different experiments were represented and averaged in the same graph. The results were expressed as mean ± SEM of the indicated *n* values.

## Results

### TAMs Induce Chemoresistance of Cancer to CDDP Through the Generation of NO

**Figure 1:**
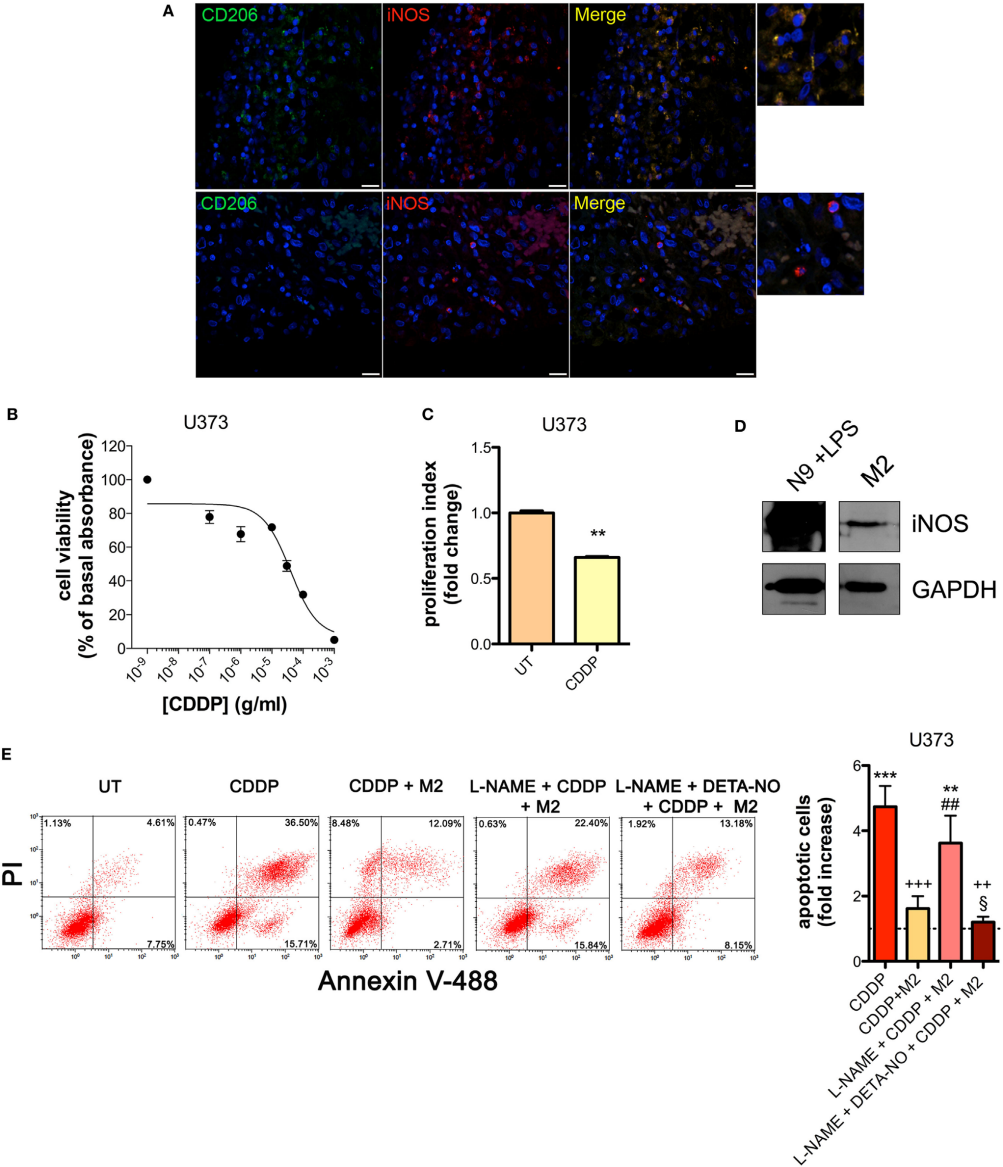
M2 macrophages protect human glioma from chemotherapeutic agent cisplatin (CDDP)-induced apoptosis through the generation of nitric oxide (NO) *in vitro*. **(A)** Immunofluorescence staining of human glioma samples with anti-CD206 antibody (green) and anti-iNOS antibody (red). DAPI (blue) was used for nuclei detection (scale bar = 20 µm). Panels on the right represent enlarged image details. **(B)** U373 cells were treated with increasing concentrations of CDDP for 24 h before the MTT assay. Data are expressed by setting the absorbance of the reduced MTT in the absence of CDDP as 100%. The data points represent the results obtained from four independent experiments. **(C)** Flow cytometry analysis of U373 cells proliferation at 24 h after treatment with CDDP (35 µg/ml) or untreated (UT). The data are represented as fold change of proliferation index compared with UT (*n* = 3). **p < 0.001 *vs* UT. **(D)** Inducible nitric oxide synthase (iNOS) expression in human M2 macrophages, assessed by western blotting. N9 cells treated with LPS were used as positive control. GAPDH was used as the internal standard. The images are representative of three independent experiments. **(E)** Evaluation of CDDP-induced apoptosis of U373 cells cultured alone or in the presence of M2. l-NAME (2 mM) and DETA-NO (20 µM) were added 1 h before CDDP (50 µg/ml, 24 h) administration. Panel on the right shows apoptosis quantification expressed as fold increase of total apoptotic cells (annexin V^+^/PI^−^ and annexin V^+^/PI^+^ cells) compared to their respective UT controls (dashed line) (*n* = 4). **p < 0.001, ***p < 0.0001 vs UT; ^++^*p* < 0.001, ^+++^*p* < 0.0001 vs CDDP; ^##^*p* < 0.001 vs CDDP + M2; ^§^p < 0.05 vs l-NAME + CDDP + M2.

The presence of M2-polarized macrophages in human glioma was assessed using sections of bioptic specimens of glioma, which were co-immunostained with the M2 subtype TAM marker CD206 and iNOS. Inside tumors we found areas with high density of cells positive for both proteins, namely M2-TAMs, and also cells positive only for iNOS, namely M1-TAMs (Figure 1A). The presence of double positive cells demonstrates that M2-TAMs in gliomas are able to produce NO.

To investigate the role of NO generated by macrophages on CDDP-induced apoptosis we chose human U373 and mouse GL261 glioma cell lines, which are defective in the expression of both constitutive (nNOS and eNOS) and iNOS enzymes (Figures S1A–C and S2A–C in Supplementary Material). As shown in the viability assay of Figure 1B, U373 cells responded to CDDP (24 h) with an IC_50_ of 38.96 ± 0.17 µg/ml, a concentration at which cell proliferation was also significantly reduced (Figure 1C). Glioma cells were co-cultured with human peripheral blood mononuclear cell-derived macrophages, polarized to M2 (Figure S3A in Supplementary Material) and expressing detectable levels of iNOS (Figure 1D). As shown in Figure 1E, the presence of macrophages protected glioma cells from CDDP (50 µg/ml)-induced apoptosis, an effect which was reversed in the presence of the NOS inhibitor l-NAME (2 mM, added to the cell culture 1 h before the administration of CDDP) (Figure 1E). The protective role of M2 macrophages on glioma cells treated with CDDP and l-NAME was restored after NO was re-added to the co-cultures using the NO donor DETA-NO (20 µM, 1 h before the administration of CDDP) (Figure 1E), that released a constant physiological flux of NO comparable to that of M2 macrophages (20, 54) (Figure S3B in Supplementary Material).

**Figure 2:**
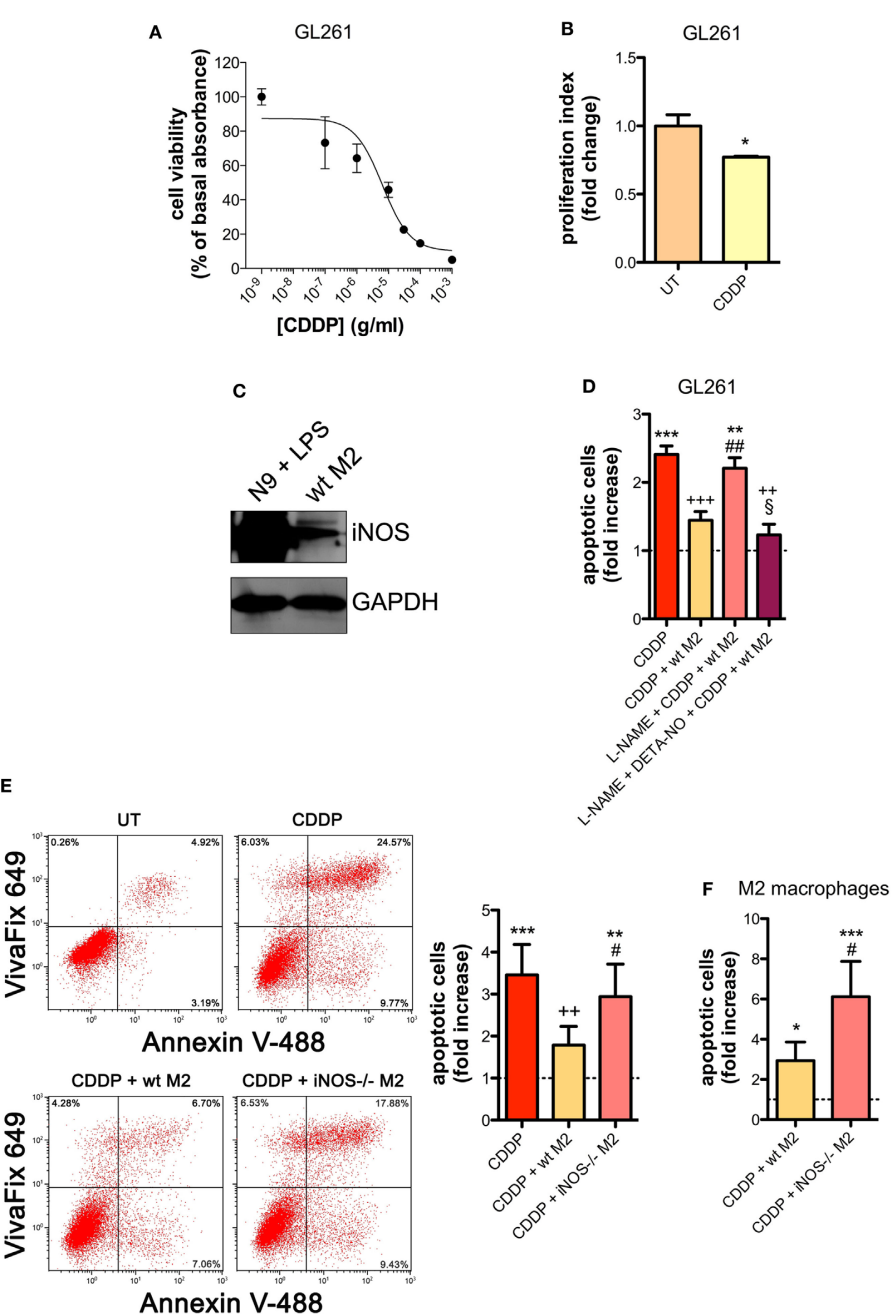
M2 macrophages protect murine glioma from chemotherapeutic agent cisplatin (CDDP)-induced apoptosis through the generation of nitric oxide (NO) *in vitro*. **(A)** GL261 cells were treated with increasing concentrations of CDDP for 24 h before the MTT assay. Data are expressed by setting the absorbance of the reduced MTT in the absence of CDDP as 100%. The data points represent the results obtained from four independent experiments. **(B)** Flow cytometry analysis of GL261 proliferation at 24 h after cell treatment with CDDP (10 µg/ml). The data are represented as fold change of proliferation index compared with untreated (UT) controls (*n* = 3). *p < 0.05 vs UT. **(C)** Inducible nitric oxide synthase (iNOS) expression in murine wild type (wt) M2 macrophages, assessed by western blotting. N9 cells treated with LPS were used as positive control. GAPDH was used as the internal standard. The images are representative of three independent experiments. **(D)** Evaluation of CDDP-induced apoptosis of GL261 cells cultured alone or in the presence of wild type (wt) M2. l-NAME (2 mM) and DETA-NO (20 µM) were added 1 h before CDDP (20 µg/ml, 24 h) administration. Apoptosis quantification is expressed as fold increase of total apoptotic cells (annexin V^+^/PI^−^ and annexin V^+^/PI^+^ cells) compared to their respective UT controls (dashed line) (*n* = 3). **p < 0.001, ***p < 0.0001 vs UT; ^++^*p* < 0.001, ^+++^*p* < 0.0001 vs CDDP; ^##^*p* < 0.001 vs CDDP + wt M2; ^§^*p* < 0.05 vs L-NAME + CDDP + wt M2. **(E)** Evaluation of CDDP (20 µg/ml, 24 h)-induced apoptosis of GL261 cells cultured alone or in the presence of wild-type (wt) or iNOS^−/−^ M2. Panel on the right shows apoptosis quantification expressed as fold increase of total apoptotic cells (annexin V^+^/VivaFix 649- and annexin V^+^/VivaFix 649^+^ cells) compared to their respective UT controls (dashed line) (*n* = 4). **p < 0.001, ***p < 0.0001 vs UT; ^++^*p* < 0.001 vs CDDP; ^##^*p* < 0.001 vs CDDP + wt M2. **(F)** Evaluation of CDDP (20 µg/ml, 24 h)-induced apoptosis of M2 macrophages from wt or iNOS^−/−^ mice. Apoptosis quantification is expressed as fold increase of total apoptotic cells (annexin V^+^/PI^−^ and annexin V^+^/PI^+^ cells) compared to their respective UT controls (*n* = 3). *p < 0.05, ***p < 0.0001 vs UT; ^#^*p* < 0.05 vs CDDP + wt M2.

Similar results were obtained with the GL261 cells. Their sensitivity to the apoptogenic action of CDDP (24 h) had an IC_50_ of 6.41 ± 0.17 µg/ml in the viability assay, a concentration that also significantly decreased cell proliferation (Figures 2A,B). GL261 cells were then co-cultured with M2-polarized macrophages derived from the bone marrow of wt mice (Figures S3C,D in Supplementary Material), that expressed detectable levels of iNOS (Figure 2C). The apoptotic cell death of GL261 cells induced by CDDP treatment (20 µg/ml) was significantly reduced in the presence of wt M2 macrophages (Figures 2D,E). Notably, the presence of l-NAME restored CDDP effects but not when cells were also pre-incubated with DETA-NO (Figure 2D). Taken together these findings indicate that NO produced by M2 macrophages protects tumor cells from CDDP-induced apoptosis. Accordingly, as shown in Figure 2E, the co-colture of GL261 cells with M2-polarized macrophages derived from the bone marrow of iNOS^−/−^ mice (Figures S3C,D in Supplementary Material) did not modify the response to CDDP of glioma cells, further confirming the key role of M2-released NO. As expected, under the same experimental conditions we observed that CDDP induced apoptosis also in M2 macrophages. However, the cytotoxic effect of CDDP was different, being iNOS^−/−^ cells significantly more sensitive when compared to wt cells (Figure 2F), thus suggesting an autocrine protective effect of NO.

**Figure 3:**
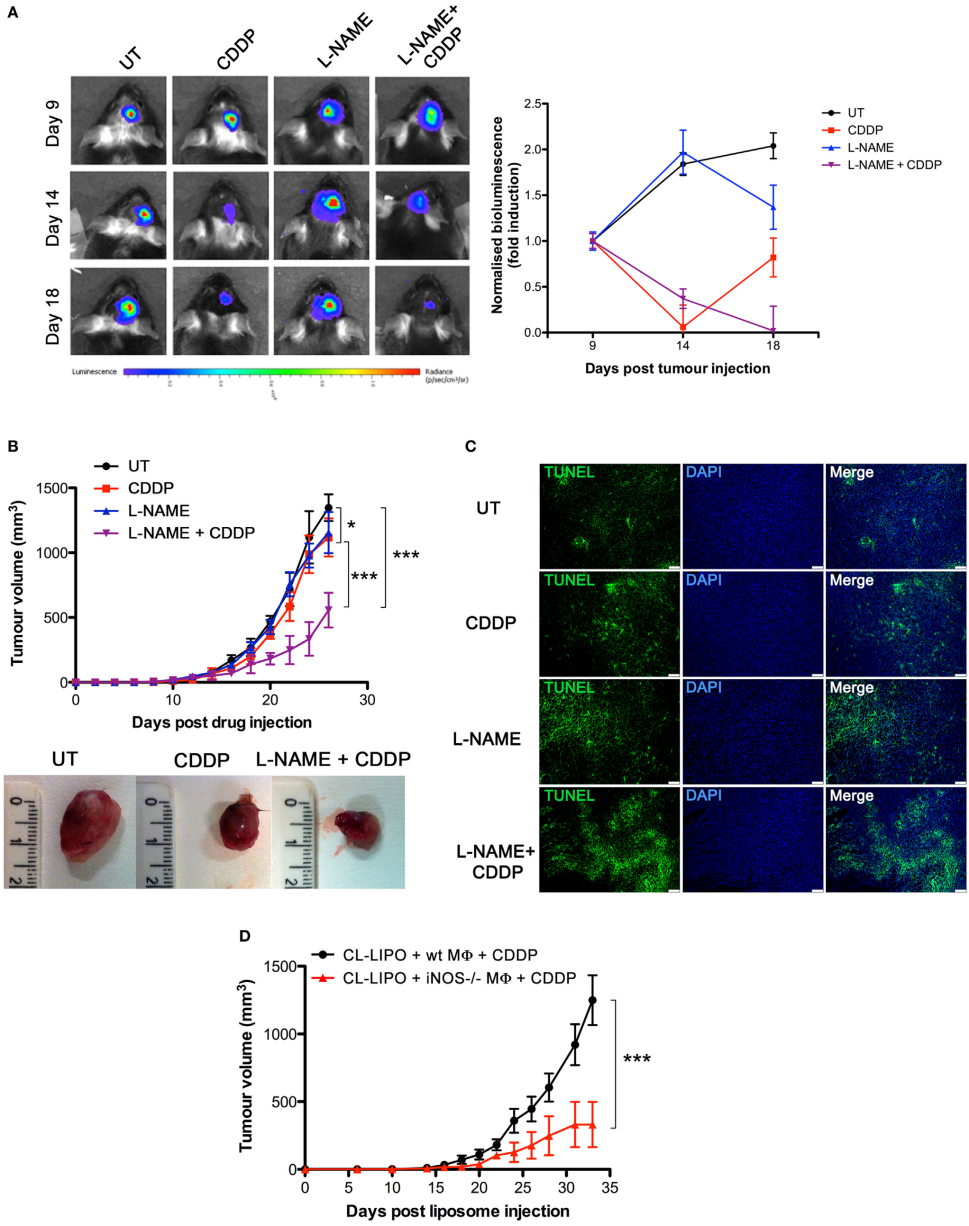
Tumor-associated macrophages protect murine cancer from chemotherapeutic agent cisplatin (CDDP)-induced apoptosis through the generation of nitric oxide (NO) *in vivo*. **(A)** C57BL/6 mice (*n* = 2–3 mice per group) were injected orthotopically in the parenchima with GL261 cells. Mice were then treated according to the scheme depicted in Figure S4A in Supplementary Material. Tumor growth was monitored by luminescence every 3 days. Panel on the left shows the bioluminescence imaging of a representative mouse for each experimental group at the indicated time points. The graph on the right shows the graphical representation of the bioluminescence expressed as fold induction compared to acquisition at day 9. **(B,C)** C57BL/6 mice (*n* = 7) were injected in the right flank with Lewis lung carcinoma (LLC) cells. Mice were then treated according to the scheme depicted in Figure S4A in Supplementary Material. **(B)** Tumor growth was monitored by measuring tumor volume (mm^3^) every 2–3 days. *p < 0.05, **p < 0.01, and ***p < 0.001 v*s* untreated control mice. The bottom panel shows the typical photographs of subcutaneous LLC allografts excised from mice at day 25 of treatment. **(C)** Representative fluorescence micrographs of TUNEL and DAPI staining of LCC excided from mice when tumor size reached ca. 500 mm^3^ (scale bar: 100 µm). **(D)** C57BL/6 mice (*n* = 7) were injected in the right flank with LLC cells. Mice were then treated according to the scheme depicted in Figure S4C in Supplementary Material. Tumor growth was monitored by measuring tumor volume (mm^3^) every 2–3 days. *p < 0.05, **p < 0.01, and ***p < 0.001 vs CL-LIPO + wild-type MΦ + CDDP group.

To investigate the role of NO *in vivo*, we carried out experiments with the syngeneic tumor mouse models generated using GL261 glioma and LLC cell lines, also these latter being defective in the expression of NOSs (Figures S2A,C in Supplementary Material), such that any effect of NO can be safely attributed to that generated in the host. GL261 (2 × 10^5^ cells) and LLC (2 × 10^5^ cells) were injected intraparenchymally (orthotopic allograft) and subcutaneously (flank allografts), respectively, in mice before i.p. administration with CDDP (4 mg/kg) both alone or in combination with l-NAME (3 mg/ml in drinking water) (Figure S4A in Supplementary Material). By monitoring tumor progression over time we found that the largest reduction of tumor growth occurred in the CDDP + l-NAME-treated group (Figures 3A,B). In addition, TUNEL assay for apoptosis detection performed on LLC tumor slices demonstrated that CDDP-induced cell death was enhanced by l-NAME administration and the ensuing inhibition of NO generation (Figure 3C). These data suggest cytoprotection as a mechanism by which NO limits the response to CDDP thus promoting tumor growth.

To assess whether macrophages in the tumor microenvironment generated the cytoprotective NO, we depleted TAMs in LLC masses by the injection of CL-LIPO in the peritumoral area (48, 55) (Figure S4B in Supplementary Material). TAMs were subsequently replaced by macrophages obtained from wt and iNOS^−/−^ mice inoculated inside LLC tumors before CDDP administration (Figure S4C in Supplementary Material). As shown in Figure 3D, mice injected with iNOS^−/−^ macrophages showed a significantly reduced tumor growth compared to mice injected with wt macrophages, indicating that NO released by TAMs is a key factor limiting tumor cell sensitivity to CDDP and thus identifying TAMs as a critical source of NO in the tumor microenvironment.

### NO Mediates Tumor Cells Resistance to CDDP Through the Generation of cGMP and the Inhibition of A-SMase Activity

**Figure 4:**
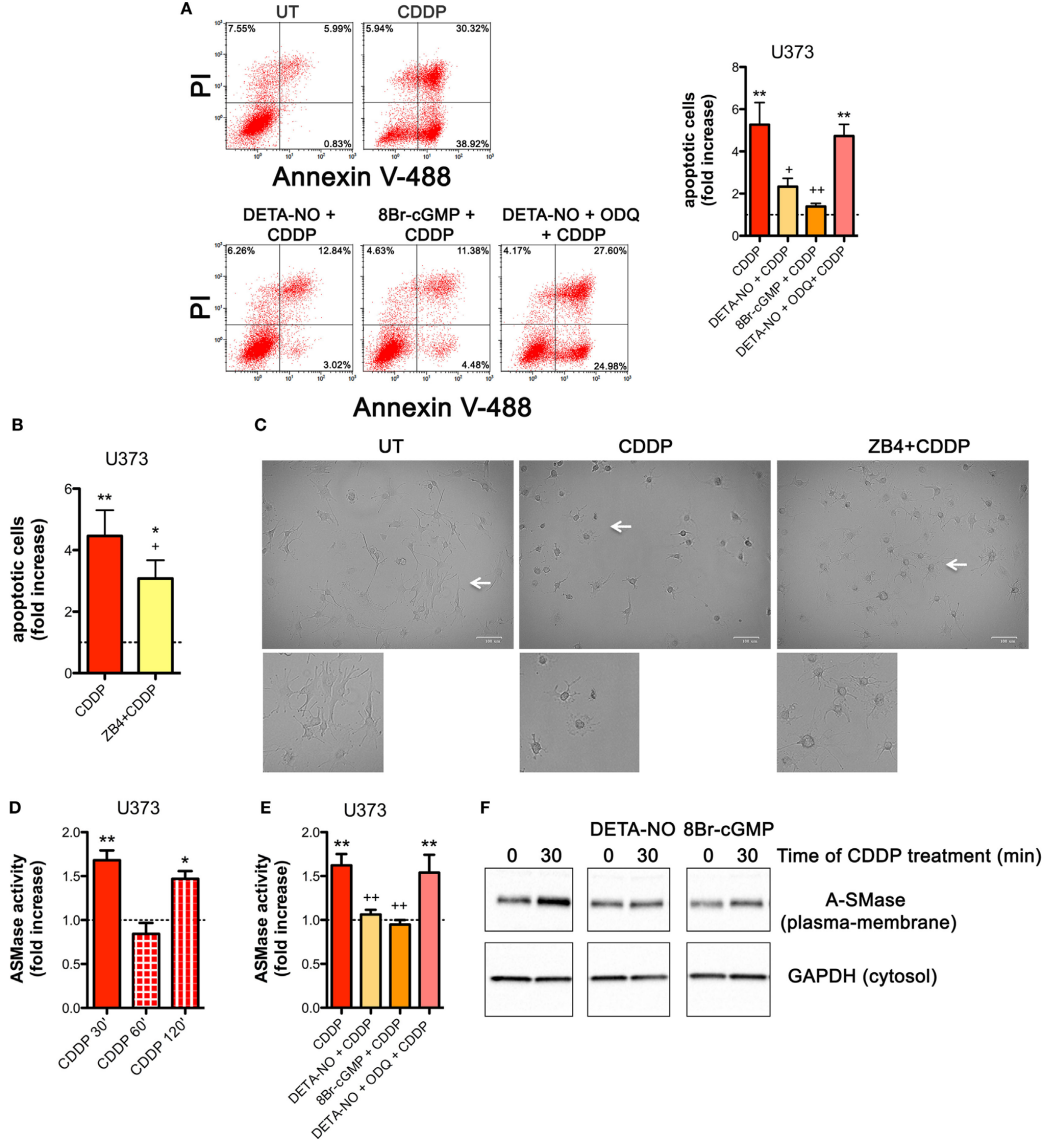
Nitric oxide (NO) mediates tumor cells resistance to chemotherapeutic agent cisplatin (CDDP) through the generation of cyclic GMP (cGMP) and the inhibition of acid sphingomyelinase (A-SMase) activity. **(A)** Evaluation of CDDP-induced apoptosis of U373 cells cultured in the presence of CDDP (50 µg/ml, 24 h) administered alone or in the presence of DETA-NO (20 µM, 1 h before CDDP), 8Br-cGMP (3 mM, 1 h before CDDP), and DETA-NO + ODQ (1 µM, 15 min before DETA-NO). Panel on the right shows apoptosis quantification expressed as fold increase of total apoptotic cells (annexin V^+^/PI^−^ and annexin V^+^/PI^+^ cells) compared to their respective untreated (UT) controls (dashed line) (*n* = 4). **p < 0.001 vs UT; ^++^*p* < 0.05, ^++^*p* < 0.001 vs CDDP. **(B,C)** U373 cells cultured in the presence of CDDP (50 µg/ml, 24 h) administered alone or together with neutralizing anti-CD95 antibody ZB4 (500 ng/ml, 1 h before CDDP). **(B)** Evaluation of CDDP-induced apoptosis expressed as fold increase of total apoptotic cells (annexin V^+^/PI^−^ and annexin V^+^/PI^+^ cells) compared to their respective UT (dashed line) (*n* = 3). *p < 0.05, **p < 0.001 vs UT; ^+^*p* < 0.05 vs CDDP. **(C)** Brightfield microscopy images representative of three independent experiments. Scale bar: 100 µm. Bottom panels represent enlarged image details marked by the white arrows. **(D)** A-SMase activity on cell lysates measured as sphingomyelin hydrolysis to phosphorylcholine at pH 5.5. Cells (*n* = 3) were treated for the indicated time points (30, 60, and 120 min) with CDDP (50 μg/ml). Enzyme activity is expressed as fold increase compared to UT (dashed line). *p < 0.05, **p < 0.001 vs UT. **(E)** A-SMase activity on cell lysates derived from cells cultured in the presence of CDDP (50 μg/ml, 30 min) administered alone or together with DETA-NO (20 µM, 1 h before CDDP), 8Br-cGMP (3 mM, 1 h before CDDP), and DETA-NO + ODQ (1 µM, 15 min before DETA-NO) (*n* = 3). Enzyme activity is expressed as fold increase compared to UT controls (dashed line). **p < 0.001 vs UT; ^++^*p* < 0.001 vs CDDP. **(F)** A-SMase translocation was evaluated, at the indicated time points after CDDP (50 μg/ml) administered alone or together with DETA-NO (20 µM, 1 h before CDDP), 8Br-cGMP (3 mM, 1 h before CDDP), and DETA-NO + ODQ (1 µM, 15 min before DETA-NO), by assessment of biotinylated plasma membrane A-SMase, using the A-SMase antibody and assessing cytosolic GAPDH expression in parallel as internal control. The images are representative of three independent experiments.

NOS/NO pathway induces many of its action *via* activation of guanylate cyclase/cGMP system (25, 44, 56–59). To corroborate our hypothesis that NO protects tumor cells from CDDP-induced apoptosis and to investigate the molecular mechanism underlying this event, U373 cells, natively deficient of NOS enzymes, were pre-incubated with the NO donor DETA-NO (20 µM, 1 h) before the addition of CDDP (50 µg/ml, 24 h). As shown in Figure 4A, Annexin V staining decreased in DETA-NO treated cells when compared to control (CDDP alone), thus revealing the paracrine key role of NO in inhibiting CDDP-induced apoptosis. Pre-incubation of U373 cells with 8Br-cGMP (3 mM, 1 h), a non-hydrolyzable and cell permeant analog of cGMP, mimicked the action of DETA-NO on CDDP-induced apoptosis while the protective effects of DETA-NO were blocked in the presence of ODQ (1 µM, administered 15 min before DETA-NO), a guanylate cyclase inhibitor that prevents NO-dependent cGMP generation (60, 61). These results indicate that NO inhibits CDDP-induced apoptosis in tumor cells through the generation of cGMP.

The death receptor CD95 contributes to CDDP-induced apoptosis in cancer cells (32). In U373 cells, the pro-apoptotic effects of CDDP were inhibited, at least in part, by the presence of the neutralizing anti-CD95 antibody ZB4 (500 ng/ml, 1 h of pre-incubation) (Figure 4B), thus supporting the critical role of CD95 in our system. Similar results were obtained by brightfield microscopy revealing that the majority of cells exposed to CDDP alone, but not to ZB4 + CDDP, presented apoptotic cell morphological changes (round in shape, shrunken cytoplasm, formation of apoptotic bodies) (Figure 4C).

Acid sphingomyelinase, a valuable enzyme in cancer progression and in the sensitivity to chemotherapy (40, 46) has been previously reported to be activated by CDDP (28, 32, 62). Therefore, we first assessed the time course of A-SMase activation by CDDP in U373 cells. Our results indicated that A-SMase activity transiently peaked at 30 min, returning to basal level at 60 min, and peaked again at 120 min after CDDP administration (Figure 4D). One of the way through which NO protects cancer and normal cells from apoptosis induced by different stimuli, including CDDP, is through the modulation of A-SMase (25, 26, 57). Since NO protective effects against apoptosis may occur within few minutes after death stimuli (56), we analyzed the effect of NO during the first phase of CDDP-induced A-SMase activation. Pre-incubation of U373 cells with DETA-NO and 8Br-cGMP strongly inhibited A-SMase activity following 30 min of CDDP stimulation, that was instead maintained if DETA-NO was added to the cells in the presence of ODQ (Figure 4E).

Commonly, A-SMase activation occurs by its translocation from cytosolic compartments to the plasma membrane (28, 62, 63). In U373 cells, cell surface biotinylation assay revealed that A-SMase specifically expressed at plasma membrane increased after 30 min of CDDP administration while this effect was reversed in the presence of DETA-NO and 8Br-cGMP (Figure 4F). Of notice, no changes were detected in the total levels (mRNA and protein) of the enzyme in cells treated with CDDP, DETA-NO, and 8Br-cGMP both alone or in combinations (Figures S5A,B in Supplementary Material). Taken together this data indicate that NO/cGMP inhibits A-SMase activation by preventing its translocation to the cell surface.

### NO Released by TAMs Reduces Synt4 Expression in Tumor Cells

**Figure 5:**
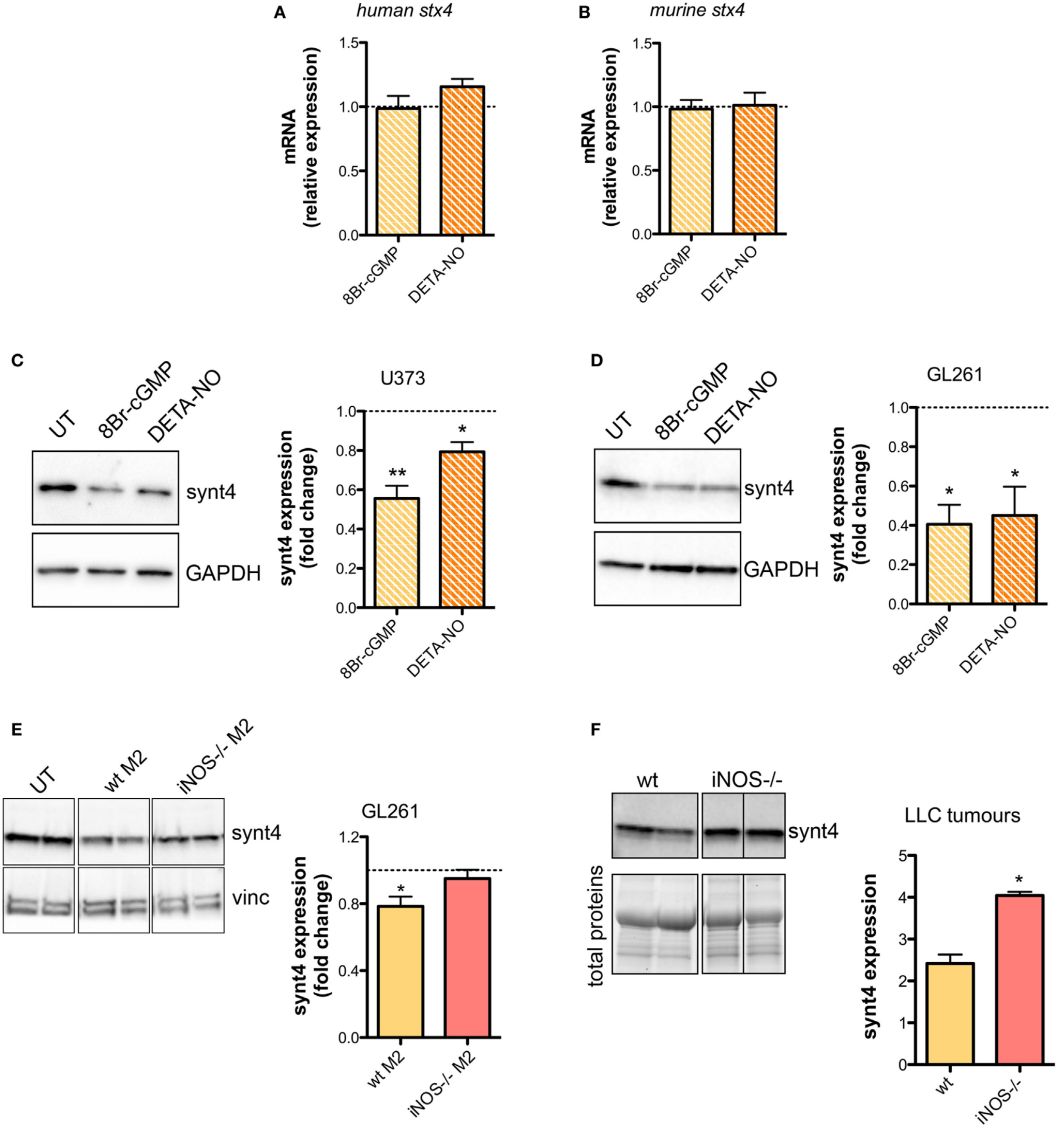
Nitric oxide (NO) released by tumor-associated macrophages reduces syntaxin 4 (synt4) expression in tumor cells. Synt4 mRNA expression in **(A)** U373 and **(B)** GL261 cells treated with DETA-NO (20 µM, 1 h) and 8Br-cyclic GMP (cGMP) (3 mM, 1 h), assessed by real-time PCR (*n* = 3). Data are expressed as the fold change over untreated (UT) controls (dashed line). Synt4 protein expression in **(C)** U373 and **(D)** GL261 cells treated with DETA-NO (20 µM, 1 h) and 8Br-cGMP (3 mM, 1 h), or **(E)** GL261 cells cultured for 24 h in the presence of wild-type (wt) M2 and iNOS*^−/−^* M2 macrophages, assessed by western blotting. The images are representative of three independent experiments. Panel on the right shows the densitometry analysis of synt4 expression. Data are expressed as the fold change over UT controls (dashed line). *p < 0.05, **p < 0.001 vs UT. **(F)** Synt4 protein expression of Lewis lung carcinoma flank allografts at 20 days after injection in wt and iNOS*^−/−^* mice, assessed by western blotting. The images are representative of 3–5 independent experiments. Panel on the right shows the densitometry analysis of synt4 expression. Data are showed using stain-free total proteins staining as loading control. *p < 0.05 vs wt.

The SNARE protein Synt4 controls the trafficking of A-SMase from the intracellular compartments to the plasma membrane and its activation upon CD95 stimulation (28) and NO/cGMP is known to regulate protein expression at both transcriptional and post-transcriptional levels (64–66). Treatment of U373 and GL261 cells with either 8Br-cGMP (3 mM) or DETA-NO (20 µM) for 1 h did not change the mRNA levels of synt4 (Figures 5A,B), thus excluding an effect at transcriptional level, still the protein amount was decreased significantly (Figures 5C,D).

Likewise in co-cultures of GL261 with M2-polarized macrophages derived from the bone marrow of wt and iNOS^−/−^ mice we found that the presence of wt M2 macrophages significantly reduced synt4 in GL261 cells when compared with control cells (GL261 alone) and with GL261 cells co-cultured with iNOS^−/−^ M2 macrophages (Figure 5E). We sought independent evidence for this effect *in vivo* in LLC flank allografts implanted in wt and iNOS^−/−^ mice, and analyzed 20 days after the tumor injection. In tumors extracts of iNOS^−/−^ mice we found higher levels of synt4 compared to those observed in tumors implanted in wt mice (Figure 5F). Together, all these data indicate that macrophage-derived NO decreases synt4 in tumors, at post-transcriptional level and in a pathway involving cGMP.

### NO/cGMP Induces Degradation of Synt4 by the Proteasome *via* PKG-Dependent Phosphorylation of Ser-78 Which Explains the Chemoresistance of Tumor Cells

**Figure 6:**
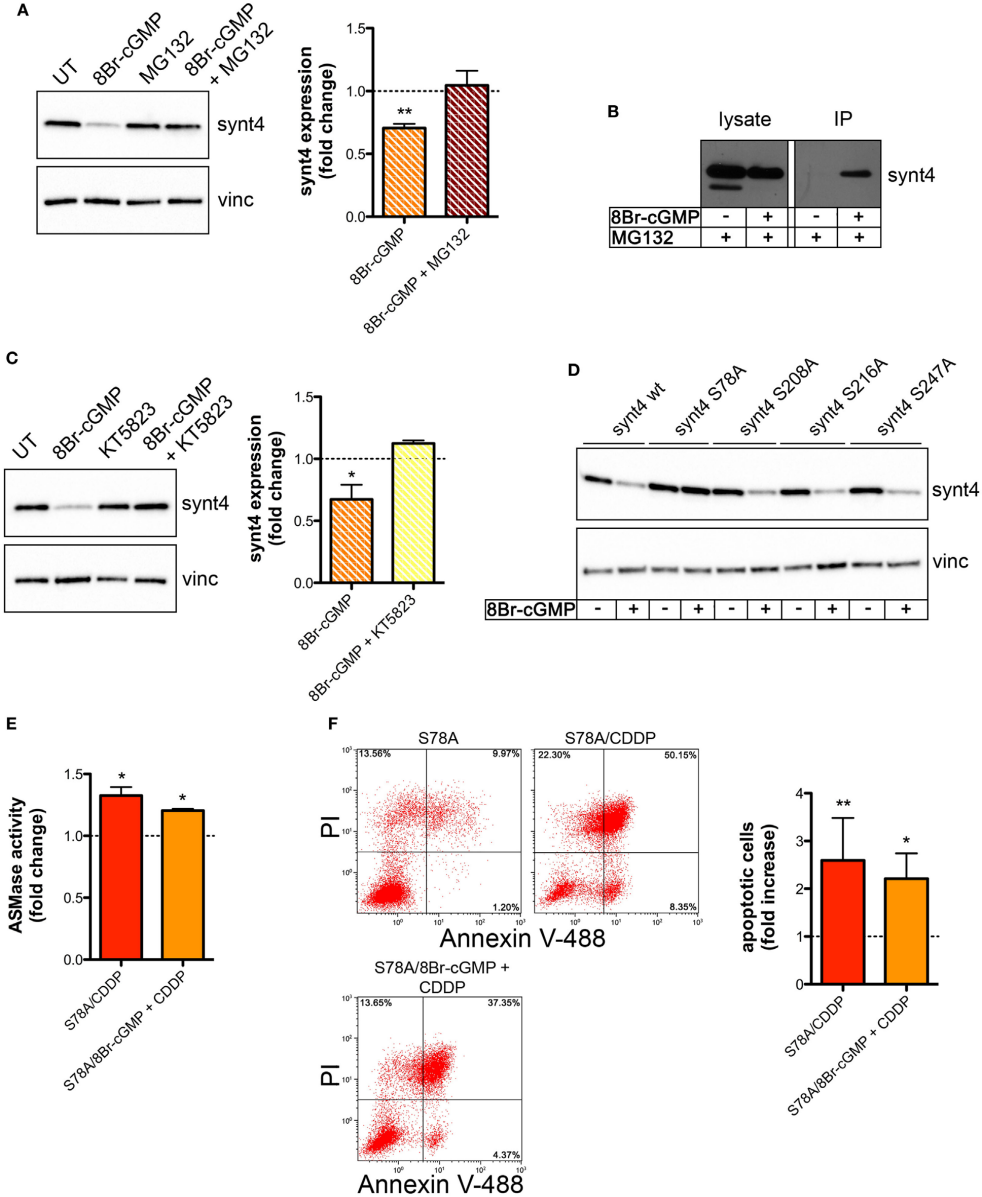
Nitric oxide (NO)/cyclic GMP (cGMP) induces degradation of syntaxin 4 (synt4) by the proteasome *via* protein kinase G-dependent phosphorylation of Ser-78 which explains the chemoresistance of tumor cells. **(A)** Synt4 expression in U373 cells treated with 8Br-cGMP (3 mM, 1 h) in the presence or in the absence of MG132 (10 µM), assessed by western blotting. The images are representative of three independent experiments. Panel on the right shows the densitometry analysis of synt4 expression. Data are expressed as the fold change over their respective controls (UT or MG132 alone) (dashed line). **p < 0.001 vs UT. **(B)** Immunoprecipitation of ubiquitinated synt4. Cells were treated with 8Br-cGMP (3 mM, 1 h) in the presence of MG132 (10 µM, 2 h before 8Br-cGMP administration), lysed, and incubated with Agarose-TUBEs to immunoprecipitate ubiquitinated protein. Synt4 expression was detected by western blotting. The images are representative of three independent experiments. **(C)** Synt4 expression in U373 cells treated with 8Br-cGMP (3 mM, 1 h) in the presence or in the absence of KT5823 (1 µM, 15 min before 8Br-cGMP administration), assessed by western blotting. The images are representative of three independent experiments. Panel on the right shows the densitometry analysis of synt4 expression. Data are expressed as the fold change over their respective controls (UT or KT5823 alone) (dashed line). **p < 0.05 vs UT. **(D)** Synt4 expression in cells transfected with synt4 wild-type and synt4 mutant proteins. GAPDH was used as the internal standard. The images are representative of three independent experiments. **(E)** Acid sphingomyelinase activity on cell lysates derived from cells transfected with the mutant protein S78A and treated with chemotherapeutic agent cisplatin (CDDP) (50 µg/ml, 30 min) alone or in the presence 8Br-cGMP (3 mM, 1 h before CDDP administration) (*n* = 3). Enzyme activity is expressed as fold increase compared to UT controls (dashed line). **(F)** Evaluation of CDDP-induced apoptosis of U373 cells transfected with the mutant protein S78A and treated with CDDP (50 µg/ml, 24 h) alone or in the presence 8Br-cGMP (3 mM, 1 h before CDDP administration). Panel on the right shows apoptosis quantification expressed as fold increase of total apoptotic cells (annexin V^+^/PI^−^ and annexin V^+^/PI^+^ cells) compared to their respective UT controls (*n* = 4).

We then explored whether the post-translational reduction of synt4 by NO/cGMP was due to an action on the proteasomal pathway. U373 cells were treated with 8Br-cGMP (3 mM) for 1 h in the absence/presence of the proteasome inhibitor MG132 (10 µM). As shown in Figure 6A, MG132 reversed the degradation of synt4 induced by 8Br-cGMP. We also found that synt4 was ubiquitinated upon 8Br-cGMP treatment (Figure 6B), thus indicating that the reduction of synt4 expression was mediated by NO/cGMP through a proteasome-dependent degradation.

The PKG, a classical signaling molecule activated by NO/cGMP, is involved in the inhibition of A-SMase activity (25) and serine phosphorylation is known to regulate protein degradation (67–70). We found that the effect of 8Br-cGMP on synt4 levels of U373 cells was inhibited in the presence of KT5823 (1 µM), a well-known PKG inhibitor (Figure 6C). We thus analyzed the human and murine synt4 primary sequence using the NetPhos phosphorylation prediction algorithm, identifying potential PKG phosphorylation residues located either at the NH_2_-terminus regulatory domain (Ser-78) or in the t-SNARE domain (Ser-208, Ser-216, Ser-247). The serine residue of each site was mutated to alanine by site-directed mutagenesis. The effect of 8Br-cGMP on the degradation of the mutants, transiently overexpressed in U373 cells, was then evaluated. As shown in Figure 6D, the mutation of Ser-78 (S78A) conferred resistance to cGMP-induced synt4 degradation, while none of the other mutations had any significant effect, thus indicating that phosphorylation of Ser-78 site of synt4 protein is required for PKG action.

Finally, we investigated CDDP-induced A-SMase activity and apoptosis in U373 cells transfected with the synt4 S78A mutant. Cells expressing this mutant remained sensitive to CDDP (50 µg/ml) even in the presence of 8Br-cGMP; indeed, the administration of 8Br-cGMP did not alter the effect of CDDP in terms of A-SMase activity and apoptosis (Figures 6E,F). Taken together, our data indicate that synt4 phosphorylation at Ser-78 and its ensuing degradation by NO/cGMP/PKG is indeed responsible for the resistance of tumor cells to CDDP *via* A-SMase inhibition.

## Discussion

In this study, we report a new mechanism at the basis of resistance of solid tumor of various origin to chemotherapeutics through an action on A-SMase. This event is based on NO produced by M2-like macrophages in the tumor microenvironment that decreases the sensitivity of tumoral cells to CDDP-induced apoptosis. We also identified in the degradation of the SNARE protein synt4 and in the ensuing inhibition of A-SMase plasma membrane exposure the molecular mechanism of NO action.

The effect of NO on tumor biology has been largely explored and has been demonstrated to be dichotomous, since it can either stimulate tumor cells growth or promote their death depending on its concentration and origin (21–24, 71). Administration of exogenous NO may directly kill cancer cells or act as chemosensitizing agents (72, 73), thus indicating NO-donors as potential anticancer drugs (74, 75). However, it has to be pointed out that the final activity of exogenous NO in cancer depends on its microenvironment, the type of cell exposed to the compound and its redox state, as well as the intracellular concentration and the duration of intracellular exposure to NO (74).

Another issue currently debated is the role of iNOS in cancer. Overexpression of iNOS and concomitant changes on cancer cell kinetics are demonstrated to have an anti-cancer action according to several *in vitro* and *in vivo* studies (71, 76–78). However, the observation that iNOS expression is high in a number of tumors and that this correlates with poor survival, has also led to the conclusion that induction of iNOS may somehow be related to tumorigenesis and tumor growth (79–82).

In light of this, it appears that the dual role of iNOS is strongly influenced by the cell situation and depends on the environment, with either induction or inhibition of iNOS having anti-cancer or pro-cancer potential based on tumor and cell types.

Inside the tumor mass, NO can be produced by both cancer cells and cells of the tumor microenvironment among which TAMs. In early events of tumorigenesis, TAMs show a M1-like phenotype, highly expressing iNOS that generates huge concentrations of NO, which in turn trigger tumor cell apoptosis and erase newly transformed cells (71). During tumor development, TAMs acquire a M2-like phenotype and promote tumor development possibly contributing also to resistance to chemotherapy (83, 84). It is worth mentioning that M2-like macrophages show a reduced expression of iNOS and generate NO at low, cytoprotective concentration (20). By *in vitro* experiments carried out in human and murine tumor cell models, i.e. U373 and Gl261 glioma cells, that do not natively express NOSs, and culturing these cells with macrophages derived from the bone marrow of wt and iNOS^−/−^ mice, we observed that the presence in tumor milieu of M2-like macrophages expressing iNOS renders tumor cells resistant to CDDP by protecting them from apoptosis. Moreover, analyzing cell death of wt and iNOS^−/−^ derived M2 macrophages, we found that iNOS^−/−^ cells were more sensitive to apoptosis, thus suggesting a possible paracrine/autocrine effect of NO (85). Inhibiting iNOS activity *in vitro* in both cell lines and *in vivo* in tumor allografts, by the administration of NOS inhibitor l-NAME increased the apoptotic activity of CDDP and reduced significantly tumor growth. The anti-proliferative effect of l-NAME has been demonstrated in different kinds of cancer cells *in vivo* (86–88). Our results obtained by the replacement of wt TAMs with iNOS^−/−^ TAMs in tumor allografts indicate that NO generated by TAMs is a key player in CDDP resistance.

Despite being one of the oldest chemotherapeutic drugs, CDDP is still a mainstay in the therapy of a variety of solid tumors (1). The efficacy of this drug is due to its multiple actions. Alongside its direct cytotoxic action at the DNA level, CDDP targets molecules promoting tumor expansion among which are cytoskeleton proteins, mitochondrial DNA, and plasma membrane proteins and lipids (2, 3, 62). CDDP activates also the CD95 death receptor on tumor cells and triggers A-SMase activity (26, 32, 62, 89). This leads to the activation of downstream apoptotic pathways and to a modification of the plasma membrane structure such that receptor clustering is favored and the death signal amplified (26, 32, 62, 90). Here, through an experiment of CD95 blockade, we corroborate the findings that the death receptor activation contributes, at least in part, to CDDP-induced cell death. The partial effect on the inhibition of apoptosis we observed was expected, because of the multiple ways through which CDDP may kill cancer cells (3).

We have previously reported that NO protects cells from apoptosis induced by different stress stimuli through the inhibition of A-SMase activity (25, 26, 56). Here, we demonstrated that such an action, at least in the case of CDDP signaling, is dependent on NO ability to generate cGMP in tumoral cells and to block the CDDP-induced, synt4-dependent A-SMase translocation on the plasma membrane and hence its activity (28). Furthermore, we demonstrated that this action of NO is mediated through synt4 phosphorylation by PKG on Ser-78 and this in turn triggers proteasome-dependent degradation of synt4. The phosphorylation of synt4 at Ser-78 site within the NH_2_-terminal regulatory domain of the t-SNARE we describe here stimulates protein degradation, likely inducing the misfolding of synt4 that triggers its recognition by the proteasome.

The very tight relationship between proteasome regulation and CD95-induced apoptosis (91) suggests the importance in cell death of the pathway of synt4 phosphorylation/degradation we report. This pathway may also be important in view of the role played by NO and A-SMase in the pathogenesis of cardiovascular, pulmonary, liver, and neurological diseases, as well as sepsis and infections (57, 92–95). Previous studies showed that synt4 can be phosphorylated by protein kinases A, Cα, and casein kinase II and that phosphorylation is a fundamental mechanism for synt4-dependent exocytosis, since binding of synt4 to cognate SNAREs is altered by its phosphorylation (96). Synt4 phosphorylation by protein kinase Cα promotes exocytosis of Weibel–Palade bodies in endothelial cells by dissociating the synt4/Munc-18 complex (97), whereas phosphorylation by protein kinase A inhibits synt4 binding to SNAP 23 (96). Recently, a role for synt4 phosphorylation state has been demonstrated for the regulation of membrane type-1 matrix metalloproteinase trafficking during invadopodium formation and tumor cell invasion (36, 37). Protein phosphorylation is known to regulate and maintain protein function and turnover, also by the activation of proteasome in physiological and pathological condition (67–70, 98, 99). Although the PKG-dependent phosphorylation of synt4 and the ensuing degradation had not been reported yet, it is conceivable that such event characterizes also the above biological systems in which both A-SMase activity and NO generation occur. Finally, the phosphorylation-induced degradation of synt4 by proteasome as a novel mechanism inducing chemoresistance to CDDP may be considered relevant also in the light of the recent advance in combinatorial cancer therapy based on proteasome inhibitors (100–102).

In summary, we demonstrated here that NO produced by iNOS expressed in M2-like TAMs efficiently protects cancer cells from CDDP-induced apoptosis leading to chemoresistance, such that inhibition of iNOS in TAMs is *per se* sufficient to restore the efficacy of chemotherapy. In addition, we found that NO activates cGMP/PKG pathway in cancer cells leading to phosphorylation of synt4 at Ser-78 site, an event which promotes the proteasomal-dependent degradation of synt4. Importantly, low levels of synt4 limit the CDDP-induced exposure of A-SMase to the plasma membrane of tumor cells thus inhibiting the cytotoxic mechanism of this drug. The identification of the role of this pathway in chemoresistance of tumors warrants further investigations as a means to identify new anti-cancer molecules capable of specifically inhibiting synt4 degradation.

## Ethics Statement

All studies involving animals were conducted in accordance with the Italian law on animal care N° 116/1992 and the European Communities Council Directive EEC/609/86. The experimental protocol was approved by the Ethics Committee of the Università degli Studi di Milano.

## Author Contributions

CP initiated the project, designed and supervised all the experiments, collected and processed the samples, analyzed the data, and wrote the article. DC and CDP processed the samples and analyzed the data, contributed to the experimental design, work discussion, and article writing. PR and MB contributed to the experimental design and to work discussion. LC and PR–Q performed the analyses on human macrophages. CM, LO, and GL performed the analyses on orthotopic brain tumor model. CM and ECa were responsible for immunofluorescence experiments. IR, MG, SZ, and MC contributed to sample collection, processing, and analysis of *in vitro* experiments. AC contributed to animal handling and *in vivo* experiments. EC coordinated the experimental work and contributed to article writing.

## Conflict of Interest Statement

The authors declare that the research was conducted in the absence of any commercial or financial relationships that could be construed as a potential conflict of interest.
