# Supplementary material for: Nitric Oxide Generated by Tumor-Associated Macrophages Is Responsible for Cancer Resistance to Cisplatin and Correlated With Syntaxin 4 and Acid Sphingomyelinase Inhibition
Source: Front Immunol. 2018 May 29;9:1186. doi: 10.3389/fimmu.2018.01186 (PMC5987706; doi:10.3389/fimmu.2018.01186)
Supplement: Supplementary file 1 [file Presentation_1.PDF]

Supplementary Materials

Nitric oxide generated by tumor-associated macrophages is responsible for cancer resistance to cisplatin and correlated with syntaxin 4 and acid sphingomyelinase inhibition

Cristiana Perrotta, Davide Cervia, Ilaria Di Renzo, Claudia Moscheni, Mariateresa Bassi, Lara Campana, Cristina Martelli, Elisabetta Catalani, Matteo Giovarelli, Silvia Zecchini, Marco Coazzoli, Annalisa Capobianco, Luisa Ottobrini, Giovanni Lucignani, Patrizia Rosa, Patrizia Rovere-Querini, Clara De Palma, Emilio Clementi

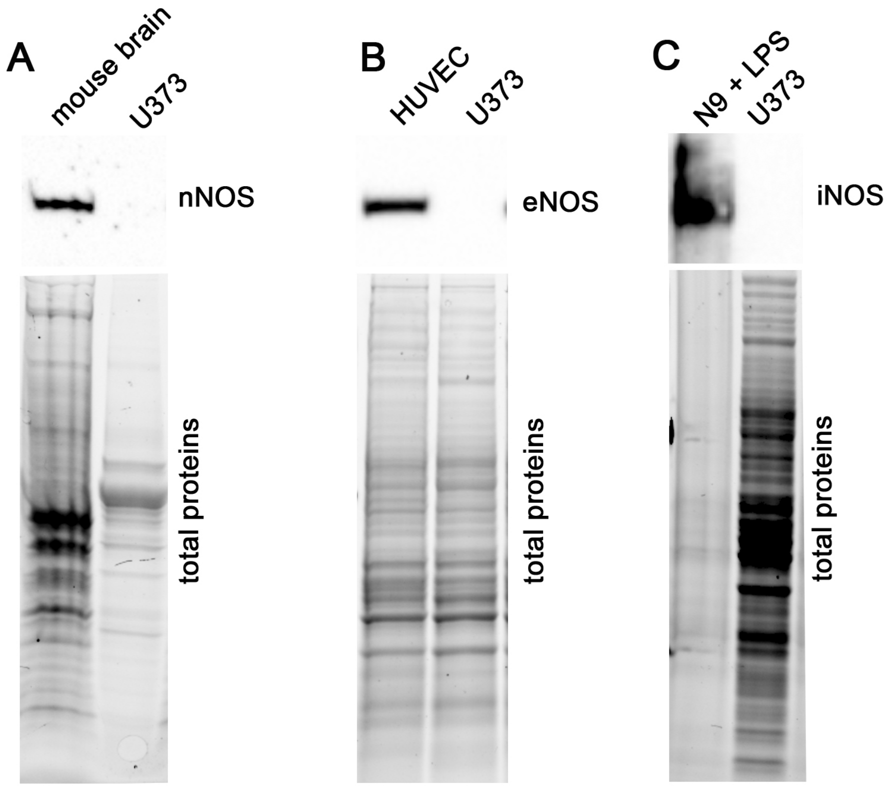

Figure S1

Figure S1. Expression of nNOS **(A)**, eNOS **(B)** and iNOS **(C)** in U373 cell lysates. Mouse brain **(A)**, HUVEC cells **(B)** and N9 cells treated with LPS **(C)** were used as positive controls. Stain free total proteins staining is showed as loading control (n = 3).

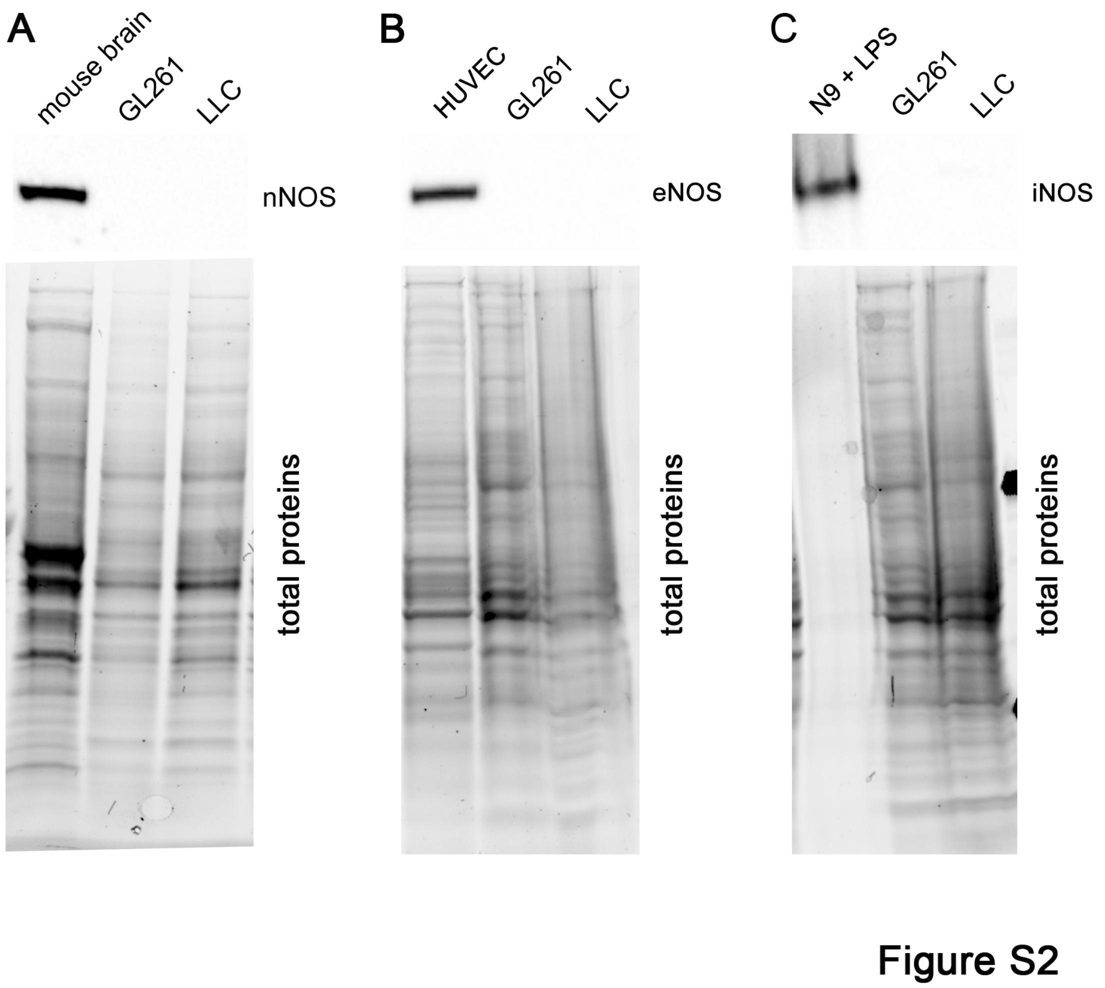

Figure S2. Expression of nNOS **(A)**, eNOS **(B)** and iNOS **(C)** in GL261 cell lysates. Mouse brain **(A)**, HUVEC cells **(B)** and N9 cells treated with LPS **(C)** were used as positive controls. Stain free total proteins staining is showed as loading control (n = 3).

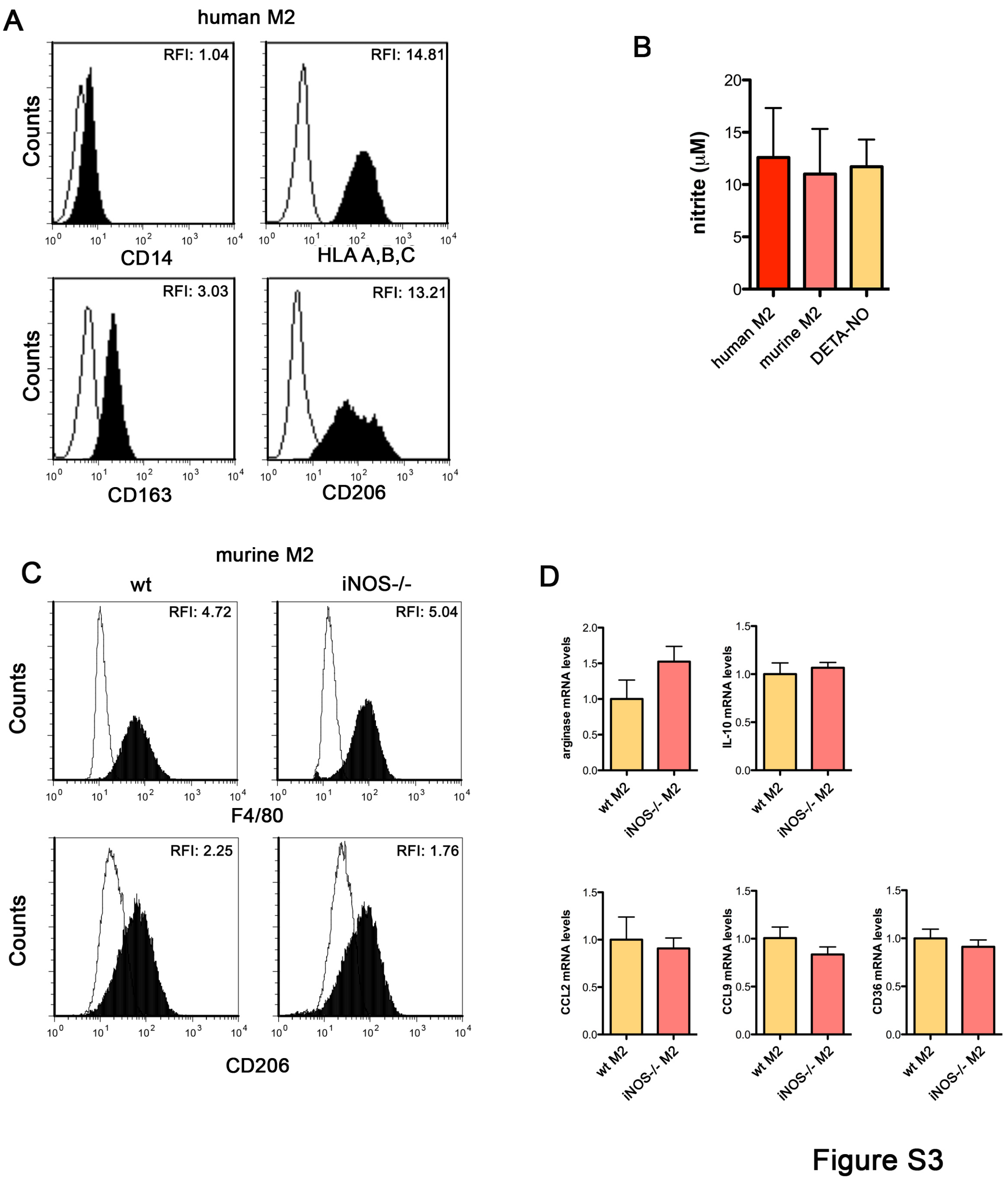

Figure S3. (A) Flow cytometry analysis of cell surface markers (CD14, HLA A,B,C, CD163 and CD206) in human M2-polarised cells. Filled histograms represent the binding of specific antibodies, whereas open histograms represent isotype-matched control antibodies. Results are representative of 3 independent experiments. **(B)** NO release by human and murine M2 macrophages and by DETA-NO was measured as nitrite production by Griess assay (n = 3). **(C-D)** Analysis of M2 macrophages derived from wt and iNOS-/- mice. **(C)** Flow cytometry analysis of macrophage cell surface markers (F4/80 and CD206). Filled histograms represent the binding of

specific antibodies, whereas open histograms represent isotype-matched control antibodies. Results are representative of 3 independent experiments. **(D)** Expression of M2 macrophage markers assessed by real time PCR (n = 3).

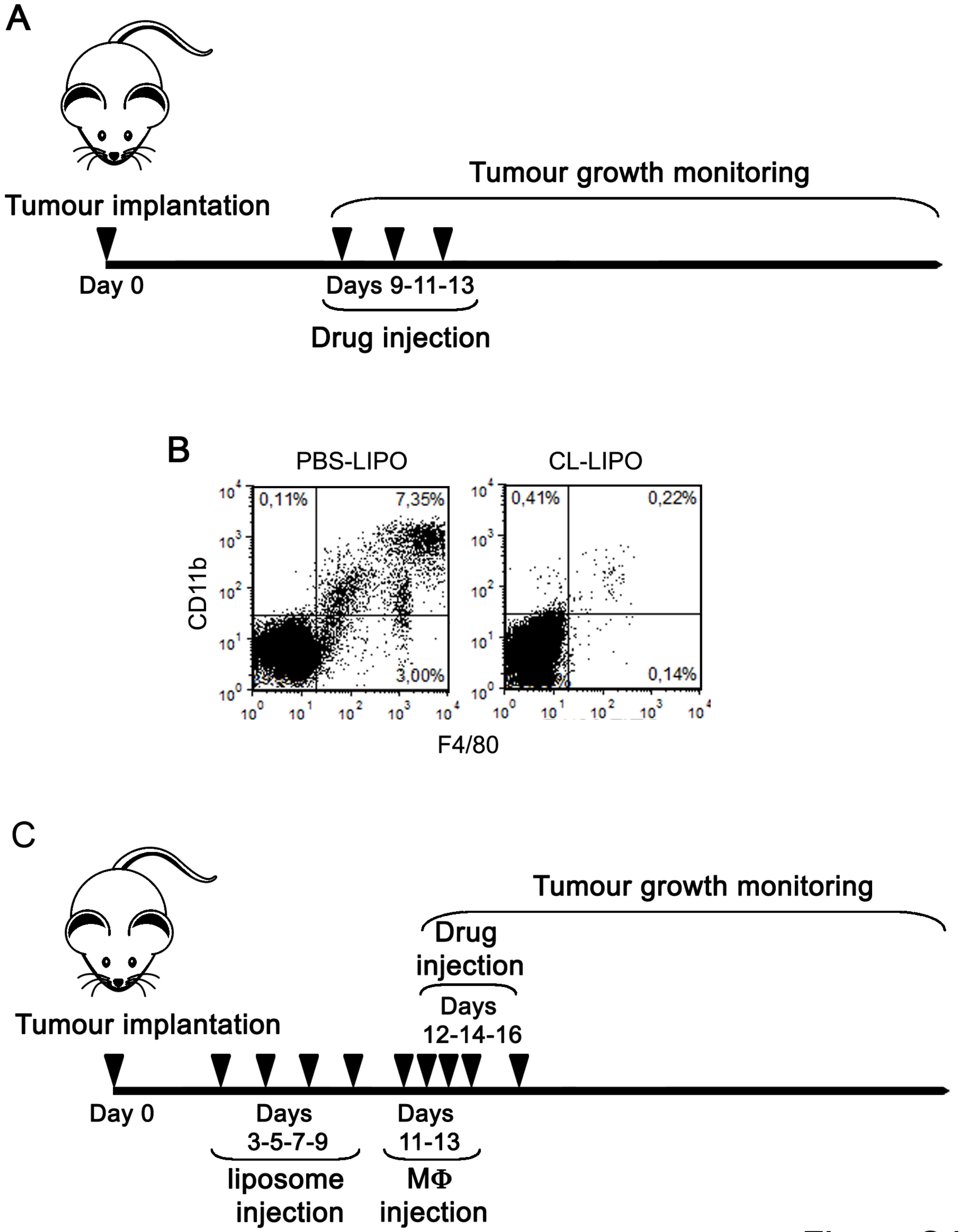

Figure S4

Figure S4. (A) Experimental scheme of orthotopic (GL261 cells) and flank (LLC cells) allografts establishment and treatment. CDDP was administered *i.p.*, L-NAME was administered in the drinking water. **(B)** Representative flow cytometry dot plot of macrophage depletion by clodronate

liposomes *vs* PBS liposomes. Results are representative of 3 independent experiments. **(C)** Experimental scheme of clodronate treatment. LLC cells were injected subcutaneously in the right flank, macrophage were depleted by clodronate liposomes and replaced by wt or iNOS-/- macrophages. CDDP was administered *i.p.*

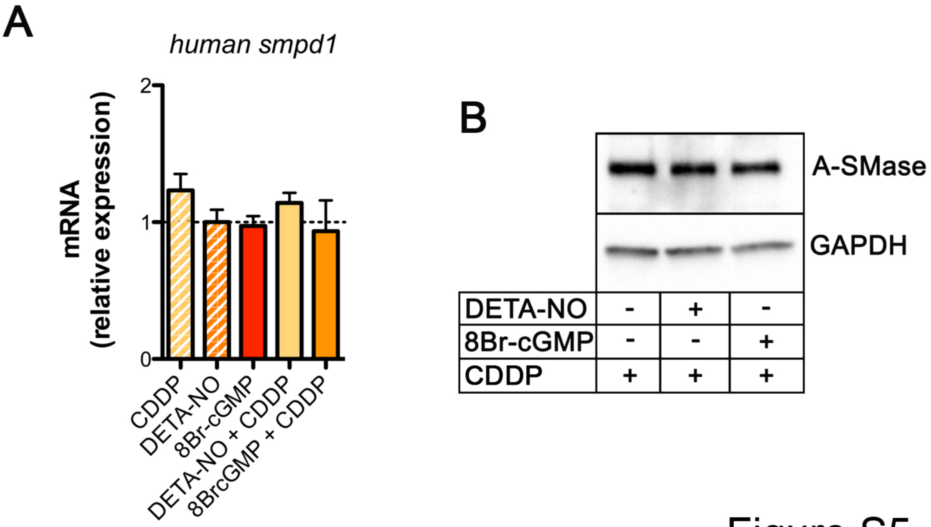

Figure S5

Figure S5. (A) A-SMase expression in U373 cells treated with CDDP, DETA-NO and 8Br-cGMP assessed by real time PCR (n = 3). **(B)** A-SMase expression in U373 cells treated with CDPP, DETA-NO and 8Br-cGMP assessed by western blotting. The images are representative of 3 independent experiments.

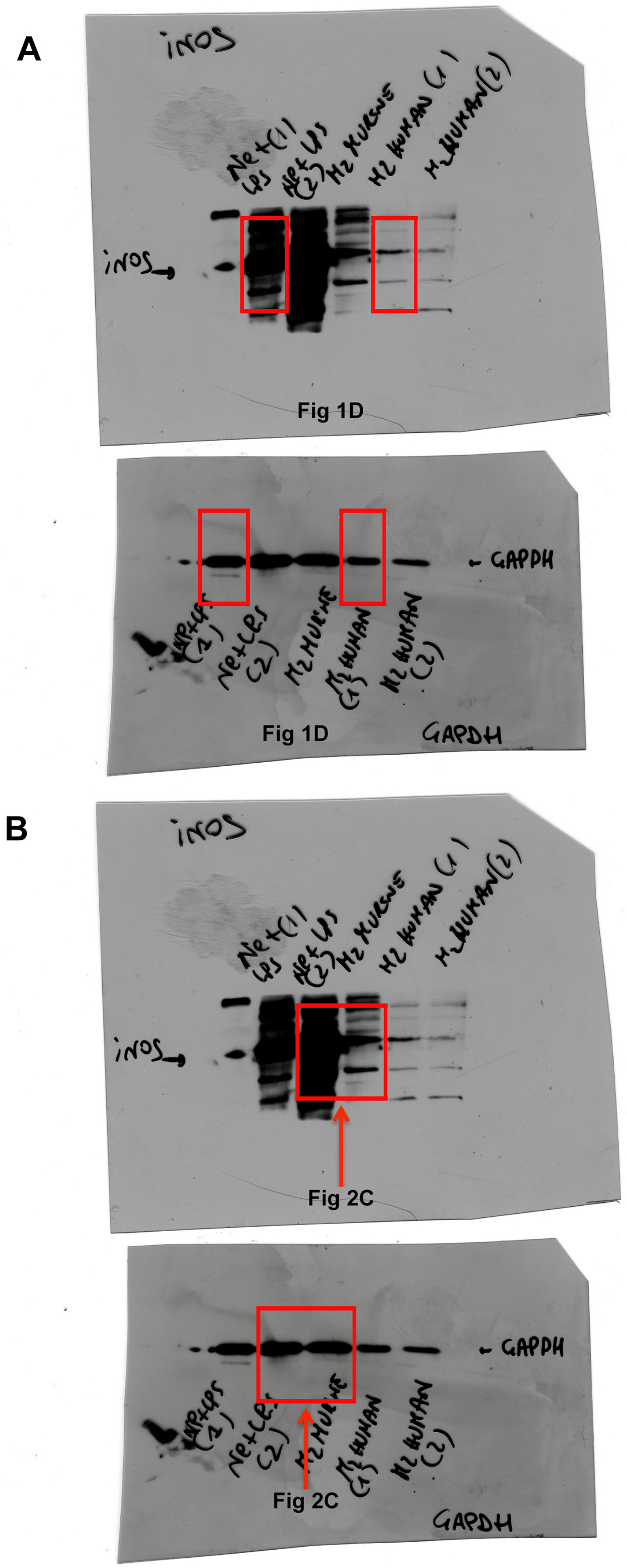

Figure S6

Figure S6. Uncropped western blotting of the images showed in Figure 1D and 2C.

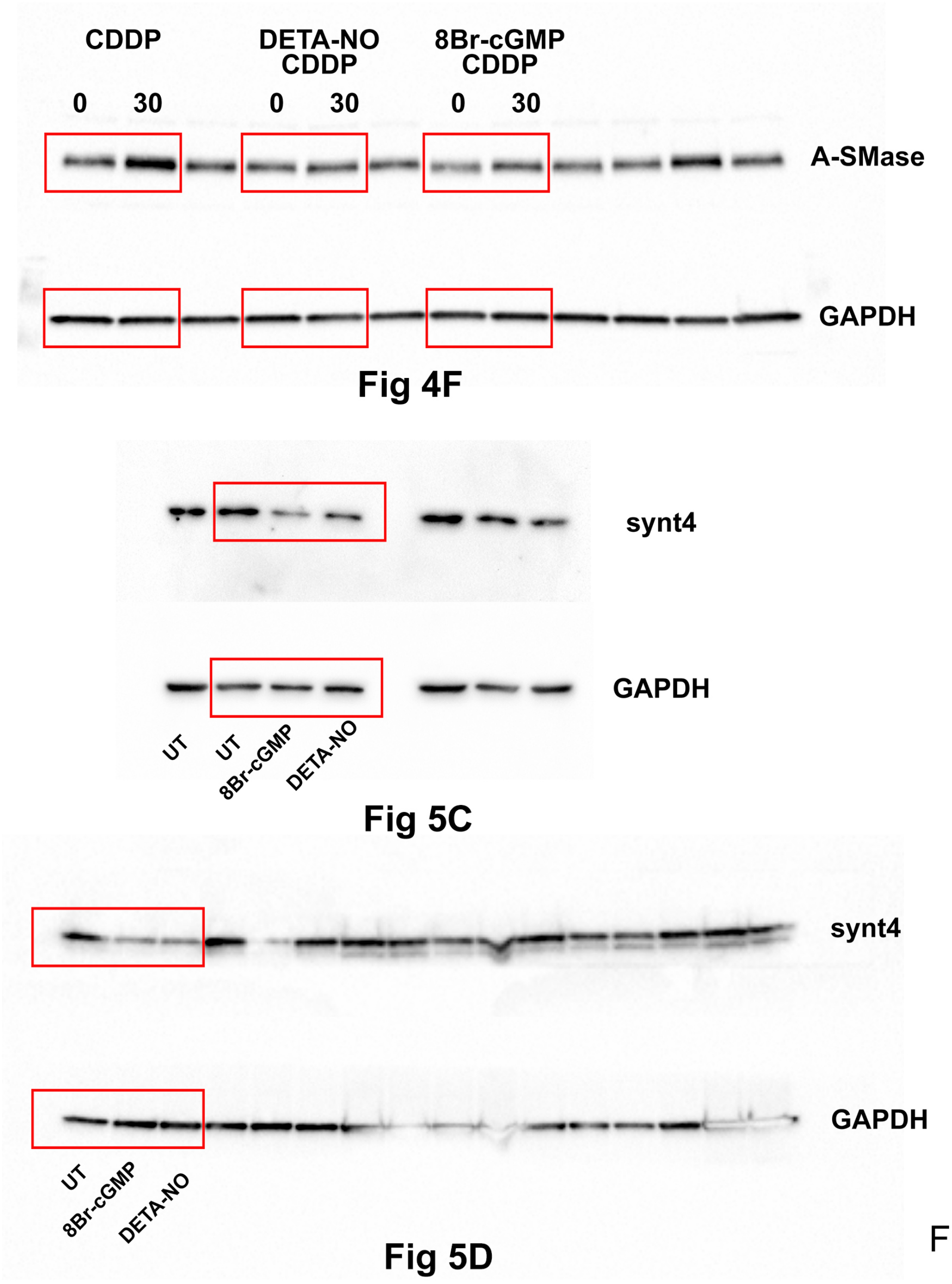

Figure S7

Figure S7. Uncropped western blotting of the images showed in Figure 4F, 5C and 5D.

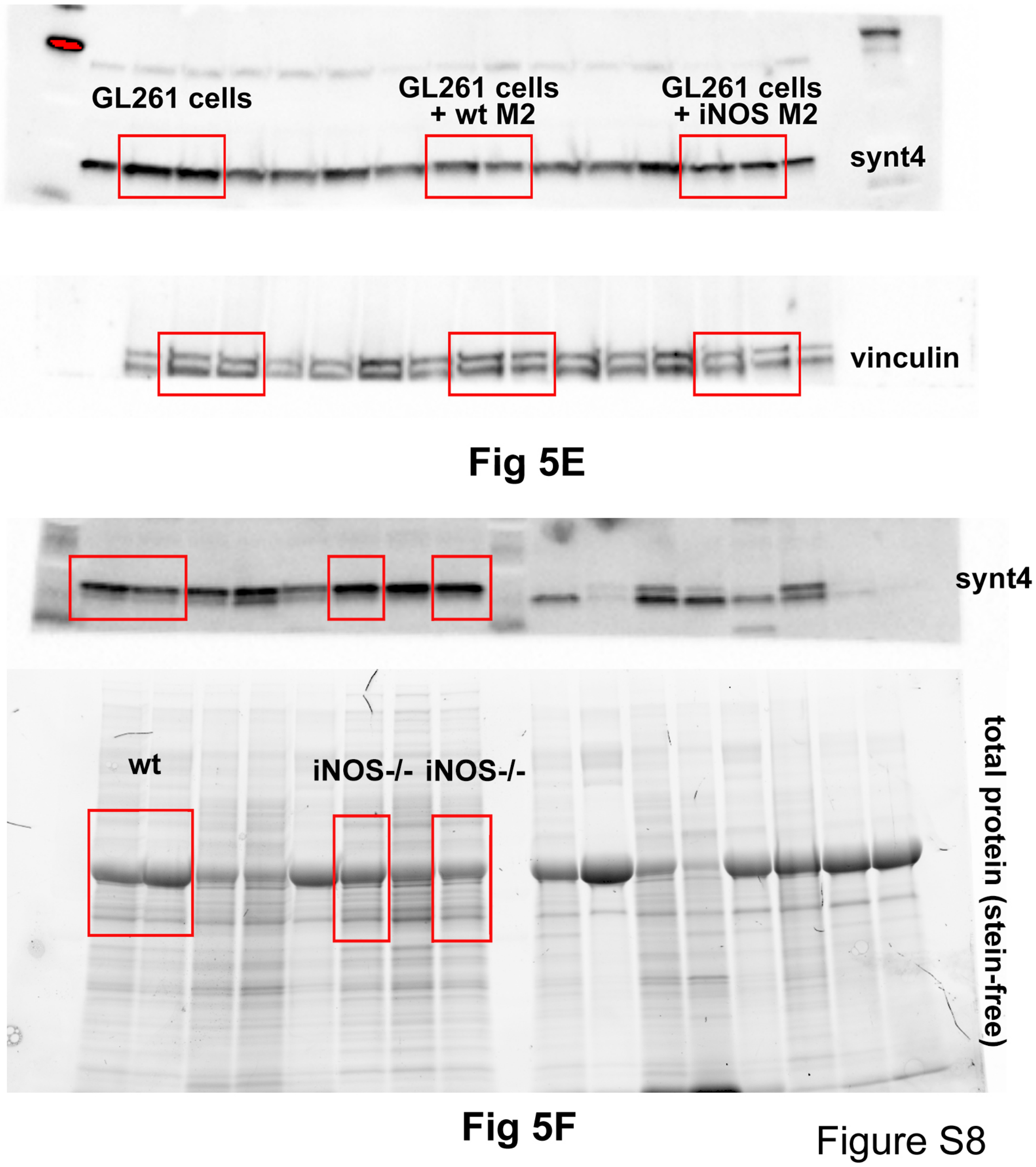

Figure S8. Uncropped western blotting of the images showed in Figure 5E and 5F.

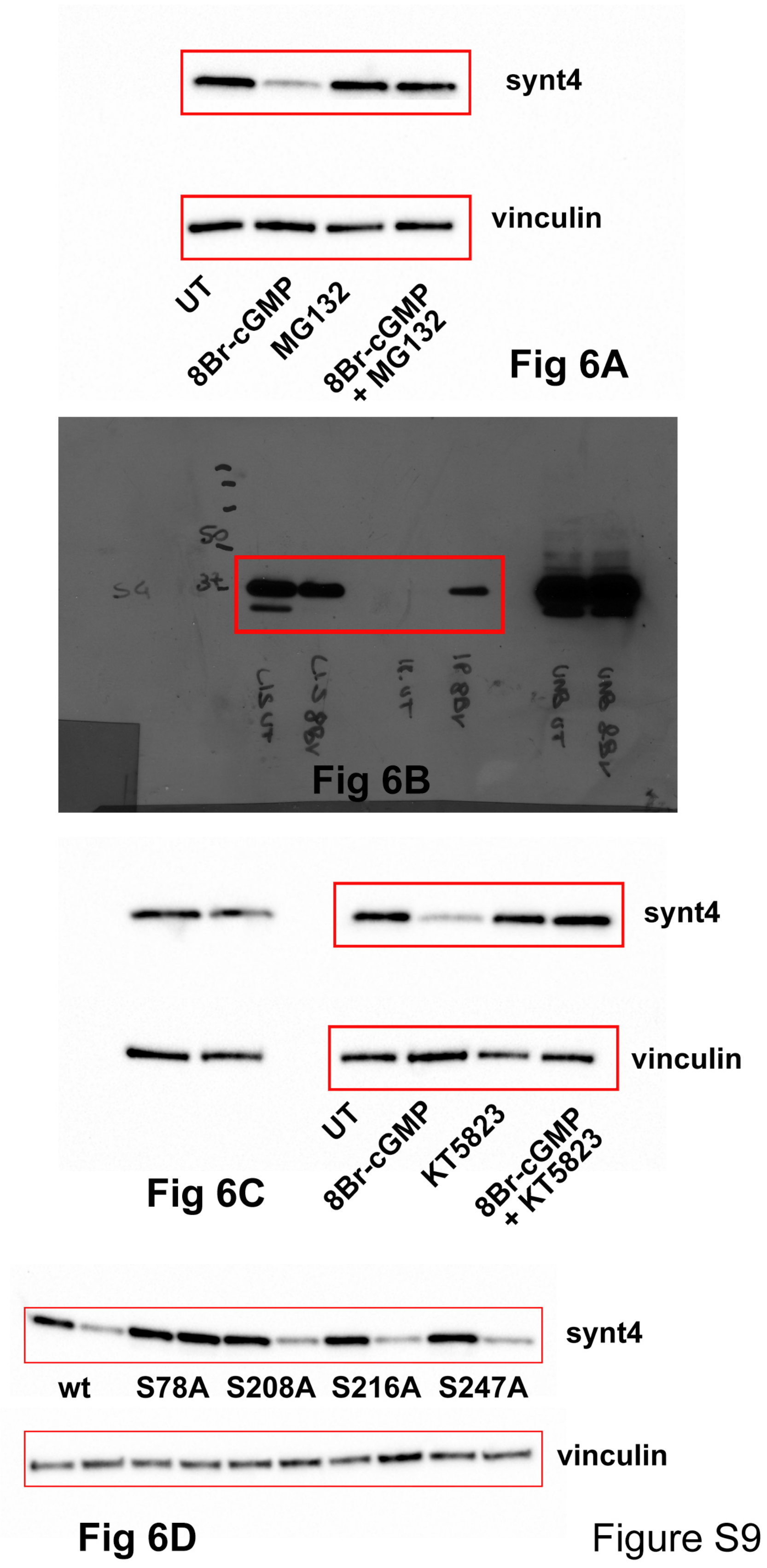

Figure S9. Uncropped western blotting of the images showed in Figure 6A, 6B, 6C and 6D.
